# Long‐Term Speech Outcomes in Moderate‐to‐Severe Childhood Speech Sound Disorder: A Systematic Review

**DOI:** 10.1111/1460-6984.70231

**Published:** 2026-04-05

**Authors:** Alexandra J. Garrett, Sermin Tukel, Angela T. Morgan

**Affiliations:** ^1^ The University of Melbourne Parkville Australia; ^2^ Murdoch Children's Research Institute Parkville Australia; ^3^ Izmir University of Economics Izmir Turkiye

**Keywords:** Childhood apraxia of speech (CAS), speech sound disorder (SSD), speech motor disorder, systematic review

## Abstract

**Background:**

Parents of children with moderate‐to‐severe speech sound disorder presenting to clinic want to understand prognosis for their child; however, there is unclear evidence as to the specific long‐term speech outcomes in this group.

**Aim:**

To review long‐term speech outcomes in children with moderate‐to‐severe speech sound disorders.

**Method:**

A systematic review identified 3697 papers; 21 papers containing 15 unique studies (six had overlapping data) met inclusion criteria for moderate‐to‐severe speech sound disorder with at least one follow‐up speech assessment unrelated to intervention outcomes. Studies were appraised for quality.

**Main contribution:**

Overall, 9 cohort studies, and 6 case studies of children aged 2y3m‐to‐11y9m at initial assessment were included. Most (11/15) had two‐to‐three follow‐up time points, and 80% (12/15) specified speech diagnosis or subtype. The most prominent moderate‐to‐severe speech diagnosis that reached criteria for inclusion was Childhood Apraxia of Speech. Speech outcomes reported in case studies included word (7/15), syllable (7/15), consonant (11/15), and vowel (5/15) accuracy, phonological patterns (9/15, prosody (2/15), consistency (1/15), and intelligibility (2/15). Case studies revealed reduced error frequency and improved single‐word accuracy and intelligibility, but persistent error types over time (e.g. token‐to‐token inconsistency). Cohort studies mostly reported on overall gross improvement, such as ‘resolved’ or ‘persistent’ disorder; however, the severity of the ‘persistent’ subgroup was not delineated. Study quality was generally good, with limitations mostly related to confounding factors such as influence of therapy, or poor or absent specification of the specific speech subtype at time zero.

**Conclusions:**

While case studies indicated reduced error frequency over time, larger‐scale data are needed to confirm findings. In the current context of health service demands for speech therapy, longitudinal speech outcome data, measuring meaningful speech outcomes are critically needed to identify predictors of longer‐term outcomes and inform prioritisation for treatment.

**WHAT THIS PAPER ADDS:**

*What is already known about this subject*

- Despite mounting evidence on the prognosis and history of mild‐to‐moderate speech and language disorder, longitudinal cohort studies of children with moderate‐to‐severe speech sound disorders are rare. This gap is notable given emerging data that severe speech sound disorders are more pervasive and pose greater risk to academic achievement and mental health.

*What this paper adds to existing knowledge*

- There are no larger cohort studies with clear granular data from which to draw conclusions. Longitudinal case studies show reduced error frequency but persistent error type in children and adolescents with childhood apraxia of speech. There are no cohort studies addressing longitudinal data for other severe speech sound disorders, such as dysarthria or fluency disorders.

*What are the potential or actual clinical implications of this work?*

- Longitudinal prospective data, with a pre‐established goal to systematically evaluate speech outcomes over time in a severe speech cohort, are critically needed to identify accurate predictors of longer‐term outcomes and inform treatment prioritisation for children with severe speech sound disorders.

## 1 Introduction

Childhood speech sound disorder involves difficulty with the perception or production of speech sounds; the smallest unit of language. Here we focus on neurodevelopmental speech sound disorders (SSD) that occur in the absence of hearing impairment, or structural anomalies such as cleft palate or macroglossia (Turner Swartz et al. 2025). There are five core speech production disorders, ranging from most to least common: articulation disorder, phonological disorder, stuttering, Childhood Apraxia of Speech (CAS), and dysarthria (Highman et al. 2025). These conditions occur due to perturbations at different neural levels, namely cognitive‐linguistic (phonological disorder), programming/planning (CAS), and execution (articulation, dysarthria) which impact speech intelligibility or naturalness (Table 1).

**TABLE 1:** Speech sound disorder classification, prevalence, and diagnostic criteria.

Speech sound disorder	Prevalence	Current Diagnostic Criteria
Articulation disorder	3.4% in 4‐year‐olds (Eadie et al. 2015)	Substitutions or distortions of the same sounds in isolation and in all phonetic contexts (e.g., a lateral lisp).
Phonological delay/disorder	3.4% in 4‐year‐olds (Eadie et al. 2015)	**Delay**: Presence of speech errors patterns typical of younger children, as determined by normative data where less than 10% of children within a six‐month age band produced the error.
		**Disorder**: Consistent (or inconsistent) use of ≥1 errors produced by less than 10% of children in any age band. (Dodd et al. 2025)
Fluency disorder (stuttering, cluttering, stammering)	0.33% children aged prep to grade 6 (McKinnon et al. 2007)	Speech characteristics (sound or syllable repetition, prolongations, blocks) (Scott Yaruss et al. 2012)
Childhood apraxia of speech	0.1% children (McKinnon et al. 2007)	Speech characterised by inconsistent errors on consonants and vowels, lengthened and disrupted co‐articulatory transitions, and disturbed prosody (ASHA 2007).
Dysarthria	0.1% 4–8 years olds (Shriberg et al. 2019)	Breathy voice, frequent pauses for inspiration, reduced precision and rate of articulation.

Common first questions parents ask when their child is diagnosed with a moderate‐to‐severe SSD include ‘will my child's SSD resolve’ and ‘when’ (Dodd 2011; Eadie et al. 2015). Previous studies have indicated that severe SSDs persist for longer, and with poorer outcomes than more common and typically milder speech difficulties (Mccabe et al. 2024; Magielski et al. 2025). Yet despite over a century of investigation of speech and language phenotypes, few studies have examined the longer‐term outcomes for moderate‐to‐severe childhood SSDs. This limits accurate prognostic counselling for parents who present to the clinic with concerns regarding their child's presentation and future. Specific to CAS, a prototypic severe SSD, recent discussions have emphasised the need for evidence‐based practice across the lifespan (Golding 2004); however, granular data on speech specific outcomes over time, are currently lacking. This limits the validity and efficacy of current and in‐development treatment options, and restricts clinicians’ ability to inform and prepare children and families for reliable predictions of future outcomes.

The presence of SSD in the preschool years has been associated with longer‐term negative impacts on language, literacy, academic achievement, and mental health (Reilly et al. 2018). These findings typically arise from follow‐up studies of common SSDs of mild to moderate severity, conducted as part of longitudinal epidemiological cohorts such as the Avon Longitudinal Study (Johnson et al. 2010) and Early Language in Victoria Study (Beitchman et al. 1999). While these large longitudinal studies did not actively exclude for severe SSD, and differentiated diagnosis at follow‐up (e.g., persistent versus resolved) the original speech diagnoses were not delineated from the outset, meaning it is not possible to fully understand those clinical trajectories. It was also difficult to determine whether the resulting ‘persistent’ subgroups were characterised as severe. Hence long‐term outcome data on moderate‐to‐severe SSD is uncommon. The majority of follow‐up cohort studies of children with moderate or severe SSD focus on longer‐term language or academic outcomes and do not specify changes in speech (Beitchman et al. 2001; Conti‐Ramsden and Durkin 2015; Veritas Health Innovation 2024). This is contradictory given the growing evidence that severe SSDs pose a different and more vulnerable long‐term trajectory than mild‐to‐moderate disorders. It is essential to more reliably predict these long‐term risks to optimise prioritisation of children for speech therapy, for planning the type and required input of service delivery, and for early prognostic counselling of parent expectations (Dodd 2011; Golding 2004). There may also be protective factors for SSDs, as identified in the language field, which could be harnessed in therapy planning to improve long‐term outcomes (Cochrane 2024).

Here we review and appraise the evidence for long‐term speech outcomes in children with moderate ‐to‐severe SSD in an attempt to systematically consolidate and better understand the current state of prognostic data available in the field. We also aimed to investigate which subtypes of severe SSD are present in the longitudinal literature. A secondary aim was to document broader long‐term outcomes such as language, literacy or school achievement in children with severe SSDs.

## 2 Method

This systematic review followed the Preferred Reporting Items for Systematic Reviews and Meta‐Analysis (PRISMA guidelines, accessed via http://www.prisma‐statement.org/Protocols/ProtocolGuidance, see Table S1). The review protocol was submitted and registered with the public registry, PROSPERO (no. CRD42024608844).

We searched MEDLINE, EMBASE, and PubMed databases using MeSH terms: (American Psychiatric Association 2013) speech sound disorder (McKinnon et al. 2007) apraxia of speech (Turner Swartz et al. 2025) speech disorder (Highman et al. 2025) cohort analysis (Dodd 2011) follow‐up (Eadie et al. 2015) clinical studies (Wren et al. 2023) longitudinal study, to capture articles from 1947 to 16 September 2024 (see Figure S1). Searches were conducted by author, and a librarian with >30 years’ experience in systematic review searches.

**Research question**: How many studies measure long‐term speech outcomes in children with moderate‐to‐severe SSD? What other long‐term outcomes are investigated (e.g., literacy outcomes)?

### 2.1 Inclusion and Exclusion Criteria

This systematic review included all original, peer‐reviewed longitudinal studies (including prospective or retrospective cohort studies, case series, and case‐control studies) which fulfilled the following criteria: (a) participants diagnosed with a moderate‐to‐severe SSD (CAS, dysarthria, or severe phonological disorder) by a speech language pathologist via a diagnostic criterion appropriate for the time of the study, (b) participants aged two to 16 years at the time of enrolment in the study, (c) speech outcomes were reported for at least two time points, with a minimum of 6 months between follow‐up assessments, (d) in English. Participants with additional speech, language, or neurodevelopmental comorbidities, such as autism spectrum disorder (ASD) or learning difficulties, were included provided speech remained the primary diagnosis and focus of the study (Reilly et al. 2018). CAS and dysarthria are rare SSDs which are arguably more severe in nature than more common speech conditions of articulation or phonological disorder (Table 1). Hence here we also incorporated the term moderate‐to‐severe SSD to encompass studies of dysarthria and/or CAS as well as studies that defined a speech outcome measure denoting moderate or severe speech performance. Historical terms (e.g., verbal dyspraxia, or developmental apraxia of speech) were also included. Studies were excluded if (a) there was no specific diagnosis or description of the SSD presented; for example, use of general terms such as ‘speech and language impairment’, (b) including participants with structural (e.g., cleft palate), sensorineural hearing impairment, or neurological disorders (e.g., cerebral palsy), (c) investigated early diagnostic markers of moderate‐to‐severe SSD, such as infant vocalisations and (d) included an intervention exposure conducted by the study authors. Non‐English studies were included in the initial database search and submitted for full‐text review if an English translation was available.

### 2.2 Study Selection and Data Extraction

All abstracts were exported to the Covidence software platform (Covidence, 2024), and duplicates were identified and removed via Covidence, then manually by the authors as required. Authors read and screened the remaining titles and abstracts for inclusion. All full texts were assessed for eligibility, and a third rater reviewed articles where consensus was required. Both authors independently extracted data from final included texts, with outcomes including sample size, age‐range, diagnostic group, methods for diagnosis, follow‐up time points, speech assessment measures, and speech outcomes (see Tables 2, 3). Study authors were contacted via email in the case of missing material and/or to request or clarify data.

**TABLE 2:** Cohort studies (NHMRC Level III‐2, ‐3) examining long‐term speech outcomes in moderate‐to‐severe speech sound disorder.

Study	Sample Size	Age at baseline (y,m)	Diagnostic Group	Other diagnoses	Diagnostic criteria	Severity	Timepoints	Follow‐Up measures	Speech change over time	Other outcomes and findings
(Lewis et al. 2004)	39	4‐6y	S (*n* = 15), CAS (*n* = 10), SL (*n* = 14)	T2, 4/10 CAS had ADHD, 2/4 learning disabilities.	CAS: ≥4 CAS features, ≥2 SD below mean for total function score for oral and speech motor control protocol. Speech development history (age of first word, motor problems, slow progress in therapy noted). S + SL: failed CAS criteria, mod‐sev SSD GFTA >1.25 SD below mean, ≥3 error types in KLPA, normal OMA, TOLDP2 <8 on ≥2 subtests for SL.	Mod‐sev: GFTA >1.25 SD below mean, ≥3 error types KLPA *note mild CAS, and CAS w/o language impairment not identified in sample	2 (preschool; 4–6y, and school; 8–10y	**T1** KLPA, oral & speech motor control protocol, TOLD‐2, GFTA, MWR, NWR, conversation speech sample (50 words) **T2** Fletcher Time‐by‐count DDK task, Syllable Rate, CELF‐R, TWS‐3, WRMT‐R, WIAT, GFTA, MWR, NWR, conversation speech sample (50 words).	8/10 CAS improvement single‐word artic. 100% persistent difficulties in syllable sequencing, NWR, conversational speech (voicing, syllable reduction, ICD, vowel errors) CAS and SL similar at T1. CAS significantly below S group on all measures.	Language, literacy, performance IQ: SL more progress in language vs CAS (9/10 CAS ≥1 SD below on CELF‐R at T2). Performance IQ lower than S and SL group for motor tasks (e.g., block design, object assembly)
(Hesketh 2004)	35	3y6m‐5y	History of mod‐sev SSD	NA, OMA typical	From original study baseline (Hesketh 2000) (Hesketh et al. 2000); mod‐sev SSD: <85 in EAT, Standard score of ≥7 on CELF‐P Sentence Structure subtest, ≥70 BPVS, Score of 6 (aged 3;6–4;3) or 7 (aged 4;3–5;0) on Raven's Coloured Progressive Matrices. Poor literacy: BASII word reading spelling standard score <85	Severity from previous intervention study via PCC. At baseline PCC range 23.83‐72.27, mean 45.2; *n* = 39 severe, *n* = 20 mod‐severe, *n* = 2 mod. Current study did not report severity of follow‐up participants.	2 (preschool; 3y6m‐5y and school; 6y6m‐7y6m)	**T1 From Hesketh 2000** Intelligibility: % change in PCC (picture naming) pre‐ and post‐therapy, PA: MAB **T2** PA: PhAB Word reading and spelling: BAS‐II Picture naming: 70 words measured by PCC, RL via BPVS	Intelligibility: progress in speech as a group (PCC mean 91.20, range 59.30‐98.28). 2/35 PCC <80 (most severe at T1). 7/35 persistent speech at T2 (PCC <90)	PA as predictor for literacy: 27/35 age‐appropriate PA & literacy. *n* = 5 ≥1SD below mean for ≥ 1 PA subtest. PA T1 best predictor for literacy T2. Type of therapy from previous study no impact on current PA. Change in PCC T1 not predictive of literacy T2.
(To et al. 2022)	82	2y3m‐6y2m	SSD (*n* = 43 no Ix. *n* = 39 Ix during study)	NA study excluded for ASD, ADHD, OMA structural deficits	1.25 SD below mean in HKCAT, OR could not produce initial consonants expected for age‐group, OR ≥1 atypical error, no intervention at time of screening, no intervention during study for *n* = 43	NA	∼4 (every 6m for 2y) or until complete initial consonant inventory	HKAT, RDLS‐C (<7 years), Narrative Test of Hong Kong Cantonese Oral Language Assessment Scale (>7years), Stimulability for misarticulated sounds in nonsense syllables. Intelligibility: ICS‐TC Expressive language: scores higher/lower than ‐1.0SD.	Median time to normalisation 6.59y for no Ix group, 7.89 for Ix group. No Ix group: larger consonant inventory T1 than Ix group (mean 13.33 vs 11.74), fewer atypical error patterns, more children who passed ICS‐TC cut‐off.	Factors impacting time to normalisation. Stimulability at T1 (time to normalisation 6.02y stimulable, 7.0y non‐stimulable), & higher ICS‐TC (time to normalisation 6.17 if passed ICS‐TC, 6.76 if failed ICS‐TC).
(Shriberg et al. 2019)	14	3y1m‐5y9m	Speech Motor Delay (SMD)	NA	Confirmed SMD: Precision Stability Index (PSI) <70% on ≥1 occasion in conversational sample available <6y. Met criteria for No SMD on ≥1 later recording OR continued to meet the criterion for SMD on ≥1 later recording.	PSI determines SMD as less severe than CD or CAS. Persistent SMD if consonant deletions and substitution >9y	Retrospective longitudinal audio‐recordings, *n* = 3 no data available between 6–9y	Conversational speech sample for PCC, II, PVC. Characteristics graded against PSI. Persistence of SMD at 6, or 9 years old.	Aims 4 & 5 (persistence and prediction) *n* = 14 T1 PCC: mean 67.7, II mean 86.8, (PCC resolved by 9y 66.6, persistent 71.6). T2 PCC not reported. 3/14 persistent after 9y Nil association between PSI% score & persistence. 11/14 resolved by 9y averaged poorer early PCC and II Nil association between magnitude of PSI and persistent SMD	Aims 1–3 (Phenotype) *n* = 50 Range of prevalence between study samples (range 2.8‐22.4%). SMD significantly lower EL, single‐word encoding, lower average words per utterance, lower PCC, PVC and II.
(Lewis et al. 2006)	38	3y‐7y	Mod‐severe SSD	NA	≥3 errors KLPA, <90% PCC from conversation sample, GFTA ≤10%ile, pass pure tone audiometric screening test, <6 past episodes of otitis media, typical peripheral speech mechanism, IQ >80 on WISC‐III	Mod‐severe score ≤10%ile GFTA, PCC <90%	2 (preschool 3–7y, school 7–12y)	**T1** conversation sample (PCC/PCC‐R), NWR, MWR, oral speech structure, function & DDK, KLPA, TOLDP2/P3, NVIQ **T2** oral motor skills via Fletcher time‐by‐count DDK, CELF3, WRMT, WIAT, TWS3, GFTA, 50‐word conversation sample (PCC/PCC‐R), NWR, MWR, NVIQ	**T2** GFTA & PCC identified; recovered (*n* = 15, PCC >96%, GFTA 99 percentile), persistent (*n* = 13 ≥3 errors GFTA and <65 percentile, <96 PCC), remainder met criteria for resolved on 1/2 measures (excluded). Higher scores on artic/phon factor associated with greater rated of recovered SSD.	Persistent SSD greater risk of reading deficits Semantic/syntactic factor higher correlation with T2 reading outcomes (comprehension) Artic/phon factors higher correlation with spelling & non‐word decoding
(Lewis et al. 2023)	32 (22/32 for developmental trajectory component of study)	3y ‐14y	CAS	NA	CAS confirmed by ASHA 2007, & (Murray et al. 2015) for earliest speech samples 26/32 enrolled between 3–6y, 4/32 7–11, and 2/32 13–14y. 22/32 selected for trajectory analysis between persistent vs resolved CAS cases (assessment ≥12y6m & speech/language assessment at 3 time points (preschool, school, adolescents)	Persistent: <16^th^ percentile GFTA and/or >4 speech errors in 10–15 min conversation on last available assessment	Retrospective, tested ≥2 times in larger CFSRS* at preschool/ school age, to adolescence & adulthood *n* = 22 CAS assessed 3x preschool, school, adolescence.	GFTA/GFTA‐2 for persistence, 10–15‐min conversation sample. Persistent vs resolved compared on the following: MWR, NWR, DDK, rapid automatised naming & elision CTOPP, single word decoding (WRMT‐R), parent report of early motor difficulties	19/32 persistent errors at last assessment (age ≥ 12.6 years). 16/19 GFTA‐2 <16^%^ile & ≥4 conversation errors. 3/19 >4 errors in conversation nil errors GFTA. No significant difference in preschool GFTA, syllable repetition, MSW, NWR, RAN, or elision in persistent vs resolved groups Resolution trajectory (*n* = 22) 6 resolved school age, 9 resolved adolescence 7 persisted in adolescence 100% continued to have difficulty with complex speech production tasks.	Persistent associated with word‐level decoding (68% had WRMT ≤85), phon. processing, MWR, motor speech sequencing, reports of fine & gross motor disorder, delayed motor milestones, early feeding difficulties & dysgraphia. Difficulties with multi‐syllable words, phon processing, literacy often present regardless of persistence/resolve of speech errors.
(Bird et al. 1995)	*N* = 31 + 31 matched for age & NVIQ	5y‐7y4m	*n* = 18 phonological impairment (P), *n* = 13 phonological impairment + language problems (PL)	NA	P: >10^th^ percentile across language measures PL: <10^th^ percentile in ≥1 measures of expressive/receptive language Typical: ≥91% PCC	48% cohort severe (PCC<50%), 29% moderate (PCC 51–64%), 23% mild (PCC 65–85%)	3 (T1 mean 5y10m, T2 7–12 months later mean 6y5m months, T3 17–24 months later mean 7y6m)	All time points: PA (not standardised), language (APT & BPVS), expressive phonology PCC via picture task (not standardised). NVIQ via WPPSI and WISC‐R. T2/T3: Reading & spelling via BAS reading subtest, Vernon spelling test	T1 PCC = 52.7% (P), 50.8% (PL) T2 PCC = 66.9% (P), 58.2% (PL) T3 PCC = 83.8% (P), 79.0% (PL)	Reading & spelling significantly lower for P/PL vs controls at T2 & T3 (letter names & sounds, non‐word reading & spelling) ∼25% P group & 50% PL group unable to spell >2 word at T3.
(Nathan et al. 2004)	47	4‐5y	*n* = 28 SSD, *n* = 19 S+L, + *n* = 47 typical controls Matching for S, SL and typical, *n* = 19 in each group.	Nil cleft lip/palate, CP, hearing impairment, associated medical condition (e.g., epilepsy), severe RLI/ELI,	Significant speech difficulties by EAT score >1SD below mean, NVIQ WNL (>85), non‐reader (raw score 0) or beginning reader (reading <7 words) on BAS word reading test. Language impairment if ≤10^th^ centile on ≥2 RL or EL measures. Monolingual English	PCC at baseline via EAT, mean score 45.6% PCC in sample at initial assessment Persistent speech difficulties EAT >2SD below mean	3 years total, assessed annually (T1 mean age 4y6m, T2 mean age 5y8m, T3 mean age 6y9m)	Input tasks: auditory discrimination real vs nonwords & picture task (T1‐T2), picture task 2 (T3) Output tasks: PCC from 20 pictures (T1‐T3), word repetition & NWR (T1, T2), extended repetition (1‐5 syllables lower frequency T2‐T3) measured by PCC. PA (not standardised) RL (TROG & BPVS T1‐T3) EL (RAPT & RBS T1‐T3) Literacy: letter name knowledge, single word reading BAS, Prose Reading NARA II, nonword reading test, spelling (T2‐T3).	T1: EAT standard score mean 73.7 (S), 57.6 (SL). Significant difference in output all time points. S group significantly worse than control at T1, SL worse than both groups T1‐T3. SL more severe and persisting difficulties. Output T1 strong predictor for output T2 + predicted input phon and language T2. 40% (*n* = 7 S, *n* = 12 S+L) persisting speech difficulties at T3 (EAT scores; mean PCC: control %, 83.47% S & SL)	Risk of literacy greater in SL group with PA deficits at 6y. Preschool language ability predictor of PA at 5y8m. Literacy problem T3: 47% S, 68% SL, 28% controls. Reading & phoneme awareness T2 critical variables for literacy T3. Predictors of spelling for S & S+L: phoneme & output (severity & persistence of speech problem), & language involvement.
(Rvachew 2006; Rvachew 2007; Rvachew et al. 2007; Mortimer and Rvachew 2010; Mortimer and Rvachew 2008)^a^	58^b^	46‐67m	Mild‐severe SSD	NA Hearing WNL, monolingual English,	<16^th^ percentile on standardised assessment Primary diagnosis speech delay, did not exclude for concomitant language disorder or CAS	Severity from GFTA‐2 percentile (%≤2 severe, 3–6% mod, 7–15% mild) *n* = 20 severe *n* = 21 moderate *n* = 17 mild	3, PreK, K, Grade 1	Expressive language: speech accuracy (GFTA‐2), phon processing, morphosyntactic analysis (PCC and MLU) & expressive language (PPVT), Phono awareness (PAT), finite verbal morphology composite, complex sentence score, reading via TOWRE	**T1 PreK** GFTA mean 4.78, PCC 65.9 (range: 40.55–91.64). (*n* = 20 sev, 21 mod, 17 mild) **T2 K** GFTA mean 12.40, PCC 75.54 (26% typical GFTA) **T3 Grade 1 (*n*** = **37** **Mortimer and Rvachew** 2010) GFTA range 0.50–62 (56%–59% below norm) PCC range 53.13–98.23 (below average in 47%–50%)	Receptive vocab and speech perception preK significantly correlated with sight word reading in Grade 1. Receptive vocab, speech perception, PAT, articulation (PCC/GFTA) correlated with non‐word decoding Grade 1. SSD preK weak nonword decoding skills at Grade 1.

Abbreviations: BAS‐II, British Ability Scales 2^nd^ Edition (Elliott et al. 1996); BPVS, British Picture Vocabulary Scale (DUNN et al. 1982); CD, childhood dysarthria; CELF, Clinical Evaluation of Language Fundamentals‐Revised (Semel et al. 1987); CP, cerebral palsy; CTOPP, Comprehensive Test of Phonological Processing (Wagner et al. 1999); DDK, diadochokinetic tasks; GFTA, Goldman Fristoe Test of Articulation (Goldman 1986); GFTA‐2, Goldman–Fristoe Test of Articulation‐2 (Goldman et al. 2012 Oct 8); HKCAT, Hong Kong Cantonese Articulation Test (Cheung et al. 2006); II, intelligibility index; MAB, metaphonological abilities battery; MLU, mean length of utterance; MWR, multisyllabic word repetition (Catts 1986); no., number; NVIQ, non‐verbal intellectual quotient; NWR, nonword repetition (Kamhi and Catts 1986); OME, oral motor exam/assessment; PA, phonological awareness; PCC, percentage consonants correct; PhAB, Phonological assessment battery (Gallagher and Frederickson 1995); PPVT‐3, Peabody Picture Vocabulary Test‐3^rd^ edition (Dunn, L., & Dunn, L. (1997). Peabody Picture Vocabulary Test‐Third Edition. Circle Pines, MN: American Guidance Services); RDLS‐C, Cantonese version of Reynell Developmental Language Scales (Reynell 1987); S, isolated speech disorder, SL, speech and language disorder, SSD, speech sound disorder, TD, typically developing; TOLD, 2, Test of Language Development Primary – 2^nd^ Edition (Newcomer and Hammill 1988), WIAT, Wechsler Individual achievement test (Wechsler 2001); WISC‐III, Wechsler Intelligence Scale for Children – 3^rd^ Edition (Wechsler 1991), WNL, within normal limits; WRMT‐R, Woodcock reading mastery tests revised.

^a^ correspondence with author Rvachew determined studies to include the same participants, treated as one cohort in this table for results. Details of each paper in Table S1.

^b^ Summary data taken for T1‐T2 from largest sample size (*n* = 58, Rvachew, Chiang & Evans 2007) See Table S1 for data from all studies. Grade 1 (T3) data from Mortimer and Rvachew (2010).

**TABLE 3:** Case studies and series (NHMRC Level IV) examining long‐term speech outcomes in moderate‐to‐severe speech sound disorder.

Study	Sample size	Age at baseline (y;m)	Speech diagnostic group	Other diagnoses	Diagnostic criteria	Severity	Time points	Follow up measures	Speech change over time	Other outcomes
(Pollock and Hall 1991)	3 (*N* = 5, *n* = 3 followed up)	8y2m‐10y9m	DAS	Y, delayed speech, family hx speech problems, non‐verbal oral apraxia, language disorder, word‐finding difficulties, academic learning difficulties, & soft neurological signs	Cluster of features associated with DAS (e.g., difficulty sequencing phonemes, inconsistency, increase in errors at length of utterance increases), typical hearing sensitivity bilaterally, NVIQ >84, articulation via TDTA, severity level 4 via Iowa Severity Rating for language & articulation.	Severe (score of 4 ‘severe’ in Iowa Severity Rating Scale for articulation & language	2 (1y apart)	111 spontaneous single word for PVC (rhotic/non‐rhotic), segmental errors, error patterns, PCC measured T1 for each consonant manner (stops, nasal, fricatives, affricates, glides, liquids, clusters)	Subject 3: Slight improvement in nonrhotic mean (0.80‐0.90), rhotic (0‐0.06), improvement in /u/ & /i/ Subject 4: Significant progress for non‐rhotic only (PVC 0.71‐0.87) Subject 5: no change to vowel system (nonrhotic PVC 0.56‐0.54, rhotic 0‐0)	NA
(Newbold et al. 2013)	4	4y	Severe & persisting speech difficulties	N, nil hearing impairment, typical oral motor structures, nil neurodevelopmental conditions, pragmatic disorder, IQ within normal limits.	Selected after T3 of previous study (Nathan et al. 2004), with persisting speech difficulties*, T1 scores <2SD on ≥2 language & auditory measures. Nathan 2004, initially included if >1SD below mean of EAT	Report presence of persisting speech & language errors = more severe prognosis	2 (∼2 years between assessment)	Picture naming EAT, Real‐word repetition (high frequency T1, low frequency) T3), NWR, 10 word connected speech in conversation/ picture description. PWC, PWP, PCC, phon error patterns (≥5 per task), phonetic inventory.	**PWC** Improved significantly in 3/4 **PWP** Significant change over time on naming & NWR in 3/4 **PCC** All changed over time in all tasks (except S2 naming task). Greatest change across tasks. For S1 & 2, phon patterns nil change at T3.	PCC best indictor of change, but no change to specific targets, PWP better measure of intelligibility & change over time. Process analysis may mask important qualitative changes.
(Stackhouse and Snowling 1992b)	1 male 1 female (10 articulation matched controls, ages 3y3m‐4y8m)	10y7m‐11y9m	Developmental verbal dyspraxia	Y hx fluctuating hearing loss (WNL in study), phon issues, auditory discrimination, segmentation & literacy delays	Met core features: clumsiness, delayed laterality, vocal tract incoordination, groping, variable production of sounds in absence of physical or neurological changes.	NA	2 (4 years between assessments, F 10;7–14;5 M 11;9–15;7)	Imitation 43 words, 43 non‐words, compared with continuous speech via 6 mini stories, EAT at T2 only	F & M made less but more severe errors than controls. Speech improved T2, same profile of speech errors, continued difficulty with novel & complex material.	Literacy in parallel study (Stackhouse and Snowling 1992a): severe segmentation & blending difficulties.
(Turner et al. 2019)	1 male	3y10m	CAS	Mod‐sev ELI, hx ‘clumsy’ motor skills (writing, riding bike); OMA found mandibular retrognathia, reduced tongue strength, involuntary tongue movement at rest.	Diagnosed against ASHA 2007. Additional difficulties with working memory & attention CAS diagnosis (4y4m) described as “frequently unintelligible”, reduced consonant inventory & reduced syllable shapes, sound substitutions & omissions (PCC = 17), distorted vowels, atypical phon patterns & inconsistent word attempts. Groping evident.	NA	17 (different assessment battery at each time‐point, final time‐point at 15y, final speech measure at 10y5m. Speech data available from 3y10m–10y5m	GFTA‐2 at 7y4m, 9y4m, 10y5m, connected speech (Rainbow passage), picture description (Cookie theft), 10 min spontaneous speech sample, DEAP consistency test, MWR/NWR, nonword memory test, CELF‐4, phon processing & memory via CTOPP, WRAT‐4 for literacy NAPLAN at 8, 10,12, 15y	PCC improved (17% 3y–95% 7y). Vowels consistent. Atypical errors variable & persistent until 7y10m. Phon processes: FCD, stopping, WSD, CR, deaffrication resolved by 7y10m Voicing, fronting, gliding persistent at 10y5m + poor MWR &NMR, inconsistency 32% PVI intensity mean close to 0 (equal stress) at 10y5m. All consonants acquired by 7y5m	Above average literacy at 14y but nonword reading, reading comprehension & spelling weakness. NAPLAN progress from 10y–12y. 15y above national average in literacy ELI WNL by 11y. RL dropped briefly at 13;0.
Le Normand, 2000 (Le Normands 2000)	1 female	5y6m	Verbal developmental dyspraxia	MRI found enlarged ventricles, thin & incompletely myelinated corpus callosum, imbalance of axial tone	Oral & laryngeal structural typical, poor intelligibility due to absence of consonants, IQ WNL	NA	2 (2‐years between assessment)	5 Language batteries (French); comprehension, lexical diversity (20 mins spontaneous speech), grammar, neuromotor assessment, PWC, PCC from word repetition	Comprehension & speech prosody appropriate T1/T2. PCC improved (9%–30%), Lexical diversity & grammar delayed at T1/T2 (MLU 1.0‐3.0), poor control of vocal tract, groping, & inconsistency at T1 & T2.	Nil change in neuromotor features
(Marquardt et al. 2004; Davis et al. 2005; Jacks et al. 2006)^a^	3 males	4y6m‐5y10m	CAS (labelled DAS prior to 2006)	P1: mild dysarthria & oral apraxia P2: history severe RLI (WNL in study) P3: short term auditory memory & metalinguistics difficulties	All participants diagnosed with CAS via ‘cluster’ of speech characteristics via Davis 1998.	NA	3 (annual follow‐up for 3 years)	Change in consonant & syllable errors, Vowel errors, token accuracy, stability, token‐token variability APP‐R, GFTA, KLPA, spontaneous speech sample (for consonant & vowel accuracy)	Inconsistent progress between & within subjects vs consistent artic progress. Inverse relationship between increased token accuracy & decreased token variability. Error variability minimal change. Increased segmental accuracy with maturation.	NA

Abbreviations: APP‐R, Assessment of Phonological Processes‐Revised (Hodson 1986); C, consonant; CR, cluster reductions; CTOPP, Comprehensive test of phonological processing (Wagner et al. 1999); DAS, Developmental Apraxia of Speech; EAT, Edinburgh Articulation Test (Anthony and Abercrombie 1971); ELI, expressive language impairment; Hx, history; ICD, initial consonant deletion; KLPA, Khan‐Lewis Phonological Assessment (Khan and Lewis 1986). Khan‐Lewis Phonological…—Google Scholar 1986); MRI, Magnetic Resonance Imaging; NVIQ, non‐verbal intellectual quotient; NWR, non‐word repetition; PCC, percentage consonant correct; Phon, phonological; PVC, percentage vowels correct; PVI, Pairwise Variability Index; PWC, percentage whole word correct; PWP, proportion whole word proximity; TDTA, Templin Darley Test of Articulation (The Templin‐Darley Tests of Articulation 1960); V, vowel; WNL, within normal limits; WRAT‐4, Wide Range Achievement Test – 4^th^ Edition (Wilkinson et al. 1993).

^a^ Marquardt (2004), Davis (2005), & Jacks (2006) studies included the same *n* = 3 male participants, diagnostic, follow‐up, & assessment procedures. Results from all three studies are combined in this table, please find individual results in Table S2. Authors contacted.

### 2.3 Quality Appraisal

Methodological quality was critically examined by two authors using the Critical Appraisal Skills Programme (CASP) for case and cohort studies, including: (American Psychiatric Association 2013) aims, (McKinnon et al. 2007) sample recruitment, (Turner Swartz et al. 2025) diagnostic criteria, (Highman et al. 2025) outcome measures, (Dodd 2011) follow‐up time points, (Eadie et al. 2015) precision of results, (Wren et al. 2023) application and implication of results (National Health and Medical Research Council (NHMRC) 2009; Kolaski et al. 2024). Two authors independently appraised each study, and met to reach consensus (see Table S2). In the case where consensus could not be reached, the third author was consulted to review papers for final inclusion. We evaluated each study's level of evidence with the National Health Medical Research Centre (NHMRC) evidence hierarchy (Campbell et al. 2020), ranging from systematic reviews of randomised controlled trials (level I) to case series with post‐test or pre‐test/post‐test outcomes (level IV). This enabled authors to rank evidence by study design, providing a gross overview of evidence quality across included studies, prior to detailed quality appraisal.

### 2.4 Synthesis

This review summarised data on speech diagnosis, sample size, age, follow‐up assessments, speech, and non‐speech outcomes (ASHA 2007, Davis et al. 1998).

## 3 Results

**FIGURE 1:**
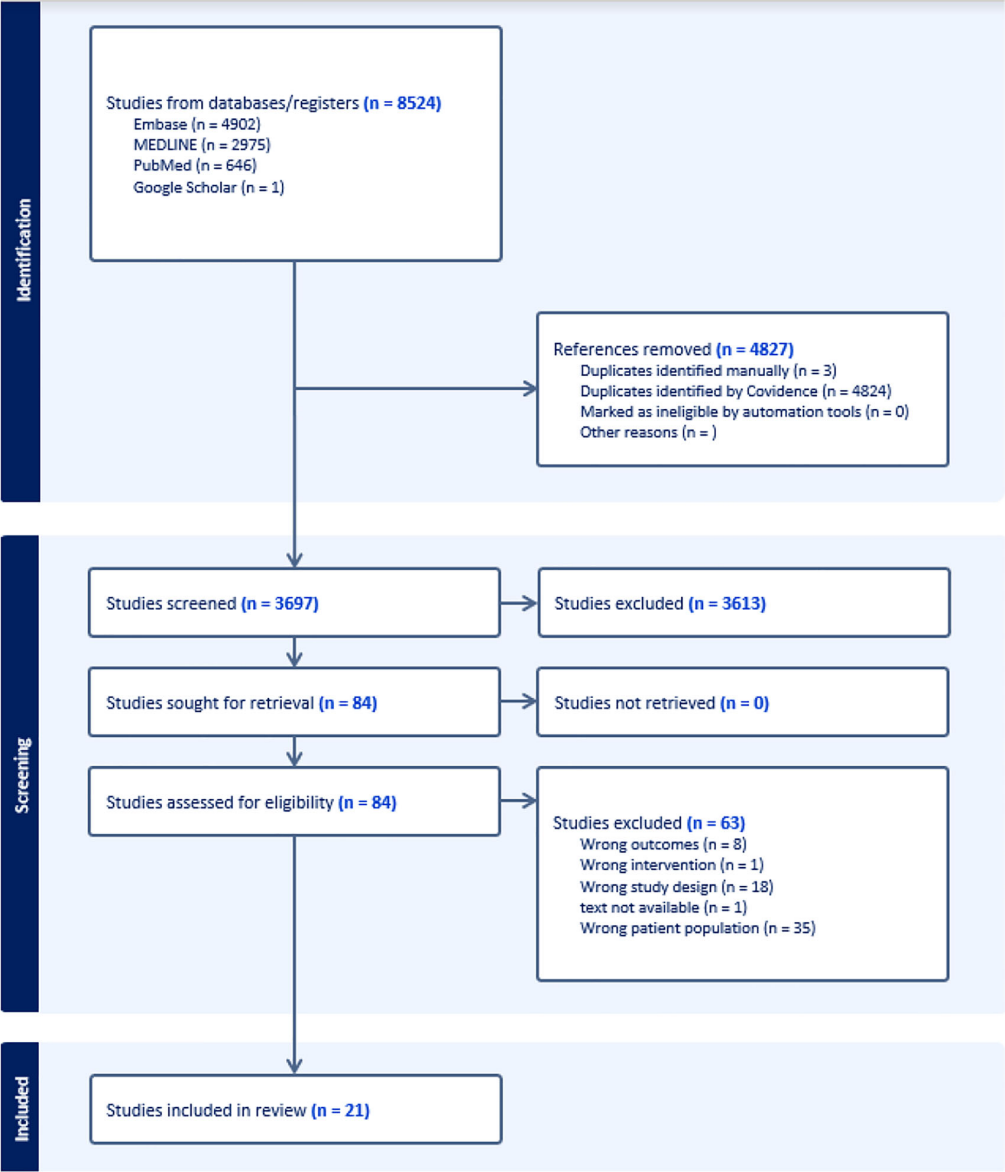
PRISMA diagram of database search from identification to final included studies. Of the 21 total included papers, five cohort studies were found to have the same sample and data (Rvachew et al., 2006–2010) and thus have been analysed as one study. Additionally three case studies used the same sample and data (Marquardt, Davis, & Jacks 2004–2006) and have been analysed as one study.

The search strategy identified 8 524 studies for review. Covidence automatically removed 4 827 duplicates, and an additional three duplicates were removed manually. A total of 3 697 studies were screened based on their titles and abstracts, and 84 full texts were subsequently screened (see Figure 1). Twenty‐one studies met inclusion criteria and were appraised for their level of evidence using the NHMRC Evidence Hierarchy which rates studies across six levels (Level I: systematic review of level II studies; Level II: randomised controlled trial; Level III‐1: pseudorandomised controlled trial; Level III‐2: comparative study with concurrent controls; Level III‐3: comparative study without concurrent controls; Level IV: case series). Quality appraisal via cohort/case specific CASP checklists found all studies met most criteria; the most frequent risk of bias included small sample size, non‐standardised speech tasks at baseline, and confounding factors (e.g., influence of prior speech therapy) (see Table S3). Two included studies featured non‐English speaking participants and assessments (To et al. 2022—Cantonese, and Le Normand et al. 2000 – French). Many studies were excluded due to involving incorrect patient populations, such as those with language or mild SSDs (Mabie and Shriberg 2017), using inappropriate study design (CASP 2018), failing to measure the relevant outcomes (Mccabe et al. 2024), or including tailored therapy strategies throughout the study, implemented by study authors (American Psychiatric Association 2013).

### 3.1 Description of Included Studies

**FIGURE 2:**
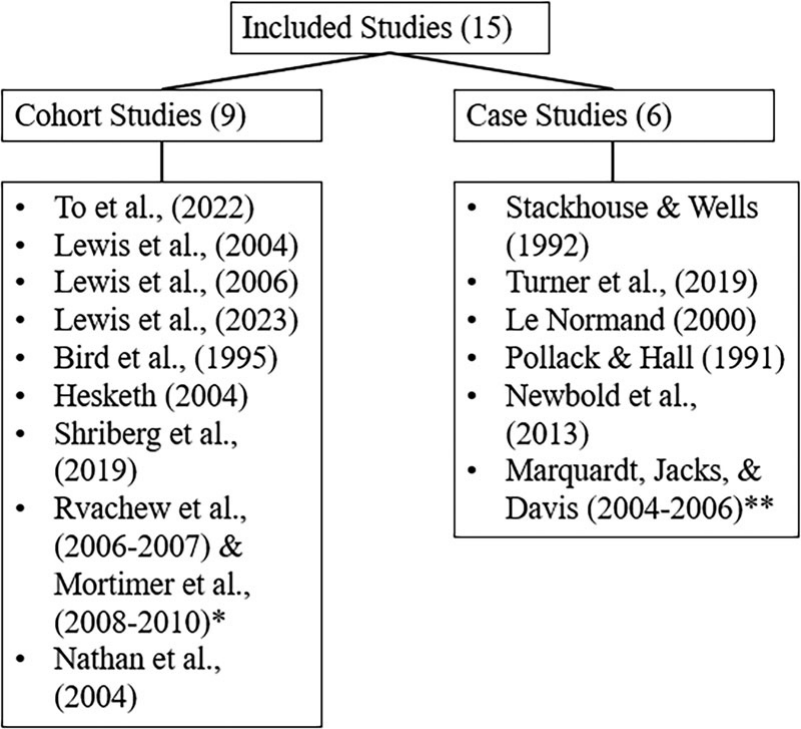
Summary of included studies. *Five papers by Rvachew et al. (2006–2007) and Mortimer et al. (2008–2010) included the same sample recruitment and data, confirmed by authors, see Table S1. **Three case studies included the same participants and data, see Table S2.

Twenty‐one papers examining speech outcomes in children with moderate‐to‐severe SSD over time were included. Yet as noted in detail later here, eight papers (3 case, 5 cohort) had overlapping samples and data (see Figure 2.). NHMRC evidence hierarchy ratings are provided in Table 2 and 3; most papers (13/21) received a rating of Level III‐2 or ‐3 equating to cohort studies with and without concurrent controls, respectively. There were eight case series (Level IV). Participant numbers varied from single case reports to cohort studies that included data from larger population study samples.

Five cohort studies were found to be attributed to the same authors and participant sample (Rvachew et al. 2006–2007, Mortimer et al. 2008–2010). Authors were contacted and confirmed these studies used data from the same original cohort. Thus, the data from these five papers were summarised together in Table 2 (see Table S4 for summary of each study). Rvachew, Evans, & Chiang (2007) was used for the speech summary data as this study had the largest sample size. Three case studies were similarly published by the same authors and included data from identical participants over identical time points (Davis, Jacks, & Marquardt 2004–2006). The outcomes of these three case studies are collated and tabulated together in Table 3 (see Table S5 for summary of each study). Thus, there were a total of 15 unique eligible studies with nine cohort (5 summarised as one), and six case studies (3 summarised as one).

Eleven studies had a primary aim to investigate long‐term speech outcomes in children and adolescents with moderate‐to‐severe SSD. Five of these included additional outcomes for literacy, phonological awareness, SSD subtype comparisons, academic achievement, and neuromotor skills. Four studies had a different primary outcome, typically literacy and/or phonological awareness in children with a history of moderate‐to‐ severe SSD; however, speech outcomes were still measured at follow‐up.

### 3.2 Diagnostic Criteria for Moderate‐to‐Severe SSD

Of the six case studies, all but one (Newbold et al. 2013) focused on children with CAS (or described with a synonymous diagnostic label). These five CAS case studies applied similar criteria for diagnosis, although diagnostic methods varied (see Table 4). In considering the current day focus on three consensus criteria for diagnosis from the American Speech‐Language‐Hearing Association (ASHA) 2007 position paper, 4/5 CAS case studies met diagnostic criteria 1 (inconsistent errors), all met criteria 2 (lengthened and disrupted co‐articulatory transitions), and three met criteria 3 (disturbed prosody) (Hall et al. 1993). Comparatively, only two cohort studies (Lewis et al. 2004, 2023) followed up a CAS cohort, with participants extracted from a larger prospective study, the Cleveland Family Speech and Reading Study (Stackhouse 1992). The remaining cohort studies reported on moderate‐to‐severe SSDs that did not differentiate motor/phonological subtypes. One cohort study examined a population retrospectively to identify the phenotype and persistence of speech motor delay (Shriberg et al. 2019). Lewis et al. (2004) applied CAS diagnostic criteria from Hall et al. (1993), Velleman and Strand (1994), Stackhouse (1992), Murray et al. (2015), Velleman and Strand (1994), and Goldman et al. (2012), which required at least four of the following features: difficulties sequencing phonemes and syllables, trial and error groping, prosodic disturbances, metathetic errors, decreased diadochokinetic rates, consonant deletions, increased error in polysyllabic words, and inconsistency. Lewis et al. (2023) retrospectively confirmed CAS diagnosis in their participants with criteria from the ASHA (2007) consensus (Hall et al. 1993), Murray et al. (2015) and Goldman et al. (2012) differential diagnosis characteristics. No eligible studies included cohorts with moderate‐to‐severe fluency disorders or dysarthria.

**TABLE 4:** Diagnostic criteria for CAS across 5 of 6 case studies.

Study	Criteria applied	Inconsistent errors on consonants and vowels	Lengthened and disrupted co‐articulatory transitions	Disturbed prosody	Other features
Pollack and Hall (1991)	(Hall 1989; Hall et al. 1990)	‘Inconsistent or variable errors…inconsistent performance’ (p. 209)	‘Difficulty sequencing phonemes…increase in errors with length or complexity of utterance…groping behaviours…slow or imprecise DDK’ (p. 209–210)	‘Inconsistent nasal voice quality…unusual prosodic patterns’ (p. 210)	‘Two and three phoneme features in error…slow progress in remediation’ (p.210) Reported nonverbal oral dyspraxia, language disorder, learning difficulties, word finding difficulties
Davis et al. (2005), Jacks et al. (2006), and Marquardt et al. (2004)	(Davis et al. 1998)		‘High frequency of consonant and syllable omissions…segmental variability’ (p. 258)	‘Prosodic abnormalities… vowel errors’ (p. 258)	
Turner et al. (2019)	ASHA consensus criteria	‘Inconsistent word attempts’ (p. 205)	‘Reduced consonant inventory…syllable shapes…sound substitutions/omissions…distorted vowels…increased errors in multi‐syllabic words and phrases…groping’ (p. 205)	‘Equal stress…prolongation errors…vowel errors…altered suprasegmental characteristics’ (p. 207)	
Le Normand (2000)	No criteria reported	‘Inconsistent articulatory output’ (p. 412)	‘Poor control of vocal tract, groping for articulatory postures…phonetic distortions, syllable reduction and difficulties with articulatory place change’ (p. 412)	NA authors note ‘appropriate intonational contour’ (p. 412)	‘Poor intelligibility—essentially due to the absence of consonants’ (p. 410)
Stackhouse and Snowling (1992)	No criteria reported	‘Variable production of sounds’ (p. 37)	‘Vocal tract incoordination, groping for articulatory postures’ (p. 37)		‘Clumsiness, delayed laterality’ (p. 37)

### 3.3 Follow‐Up Time Points

#### 3.3.1 Number of Assessment Time Points

The total number of assessment time points per study varied, ranging from two, to 17 in one case study (Turner et al. 2019). Eleven studies used two‐to‐three follow‐up points, mostly (10/11) comparing speech productions between preschool and early primary school.

#### 3.3.2 Time‐Period Between Assessments Time Points

The time‐period between assessment follow‐ups varied; only one cohort study (To et al. 2022) provided an explicit time‐period of six months between assessment points (for two years or until speech errors resolved). Seven cohort studies reported assessment at preschool and school age (approximately two years between assessment); however, the age ranges within these assessment points were broader. For example, Lewis et al. (2004) assessed children at preschool age (4‐6y), and school age (8–10y); Hesketh et al. (2004) assessed children at preschool age (3y6m‐5y) and grade 1 (6y6m‐7y6m). Two cohort studies (Shriberg et al. 2019, Lewis et al. 2023) retrospectively analysed data from participants, stipulating that audio‐recordings of collected speech data be available for two or more time points, with initial assessment prior to 6‐years‐old, and follow‐up after 12‐years‐old in the case of Lewis et al. (2023).

For case studies, 2/6 followed up participants once, with two years between each assessment (Le Normand et al. 2000 and Newbold et al. 2013). One study (Marquardt et al. 2004–2006) completed annual follow‐up assessments for three years. Stackhouse and Snowling (1992) conducted two assessments, four years apart, and Pollock and Hall (1991) reassessed 3/5 participants one year after initial assessment. Only one study, Turner et al. (2019), followed one case from 3 year 10m, to 15‐years‐old including a range of retrospective and prospective data, with a total of 17 time points.

### 3.4 Follow‐Up Assessment Measures

Follow‐up measures differed across all studies. Most studies (9/15) included a standardised articulation assessment for at least one follow‐up assessment point, and 10/15 repeated the same assessment across time points (non/standardised). The Goldman Fristoe Test of Articulation (GFTA/GFTA‐2) was the most frequently used speech assessment (40%, 6/15) (Khan and Lewis 1986; Anthony and Abercrombie 1971). Other standardised articulation and phonology assessments included the Khan‐Lewis Phonological Assessment (KLPA) and Edinburgh Articulation Test (EAT), while one older study constructed their own word list (Stackhouse and Snowling 1992; Raven 1976; Schölderle et al. 2022). The case studies by Jacks, Davis, and Marquardt (2004–2006) included the Assessment of Phonological Processes‐Revised (APP‐R), GFTA‐2, and KLPA; participants alternated assessments at each time point. Nine of 15 obtained connected speech samples; eight of which were spontaneous speech samples ranging from 5 to 20 min. Stackhouse and Snowling (1992) obtained connected speech by grouping words from the original single‐word phonology task into six mini stories where words were grouped and represented by a coloured picture. Studies with a primary focus on literacy outcomes included phonological awareness assessments, and Turner et al. (2019) included broader academic results such as national school performance testing results (the National Assessment Program—Literacy and Numeracy, NAPLAN) from early primary school to adolescence.

### 3.5 Additional Diagnoses and/or Exclusion of Secondary Diagnoses

One cohort study (Lewis et al. 2004) reported additional diagnoses (4/10 CAS diagnosed with ADHD, 2/4 learning disabilities), while 5/6 case studies reported additional difficulties. Participants (P1‐3) in Jacks, Marquardt, and Davis (2004–2006) all had a primary diagnosis of CAS, but P1 had a history of oral apraxia and mild dysarthria (resolved at follow‐up), P2 had a history of severe receptive language impairment (within normal limits at time of study), and P3 had difficulties with short term memory and metalinguistic skills. Pollock and Hall (1991) described one participant with a history of middle ear infection. Both participants in Stackhouse and Snowling (1992) experienced a history of fluctuating hearing loss. Le Normand et al. (2000) included imaging studies in their case report; MRI findings showed moderately enlarged ventricles and incompletely myelinated corpus callosum. This participant also experienced balance difficulties. Turner et al. (2019) reported their participant to have a history of ‘clumsy’ motor skills, difficulties with attention and working memory, mandibular retrognathia and reduced tongue strength. In comparison, 8/9 cohort studies excluded for additional diagnoses and all required participants to have typical hearing acuity (although only two studies reported specific scores; Lewis et al. 2006 and Lewis et al. 2023). No studies reported additional fluency disorders (e.g., stuttering or stammering).

Three cohort studies required participants have less than six episodes of otitis media before their third birthday (Lewis et al. 2004, 2006, 2023). Four studies required no neurological disorders and typical oral motor exams. Four cohort studies included IQ cut‐offs; Lewis et al. (2004, 2006, 2023) required a performance IQ ≥80; Hesketh et al. (2004) required a stanine score of 6 or 7 in the Raven's Coloured Progressive Matrices (Haas et al. 2021). Three studies did not have explicit IQ criteria but excluded for neurodevelopmental disorders.

Four case studies had exclusionary criteria. Marquardt et al. (2004–2006) required typical hearing and oral motor exam at baseline. Pollack and Hall (1991) required nonverbal IQ scores between 84 and109, and Newbold et al. (2013) excluded for hearing difficulties, structural causes of speech challenges (e.g., cleft palate), neurodevelopmental disorders, and an IQ less than ‘normal’ (cut‐off score not clarified).

### 3.6 Intervention

All cohort studies noted that their participants, or a subset of participants had undergone speech therapy prior to and/or during the study; however, the type, duration, and frequency of the therapy was not described (see Table S3). Two studies (Hesketh et al. 2004, Shriberg et al. 2019) reported their participants to have undergone targeted therapy in a previous study by the same authors (e.g., Shriberg's sample was retrospectively identified from multiple studies of participants enrolled in therapy trials for idiopathic speech delay). Only one cohort study, To et al. (2022) analysed a subset of participants (*n* = 43) who underwent no speech therapy prior to or during the study period.

Five of the six case studies reported on the type, duration, and frequency of speech therapy conducted prior to and/or during the study (Table S3), none of which was provided by study authors

### 3.7 Outcomes

Outcomes were distributed across two main categories: speech and literacy. Eleven of 15 had speech outcomes as their primary aim; however, only three cohort studies (Lewis et al. 2004; Lewis et al. 2023; To et al. 2022) and all case studies focussed on *long‐term* speech outcomes as their primary aim. The remaining cohort studies used speech outcomes to examine related aims including: factor analyses predictive of speech subtypes, literacy risk in speech versus speech and language phenotypes, and phonological awareness. Common speech outcomes included: whole word accuracy (7/15), percentage consonant correct (PCC, 11/15), syllable/segmental structure (7/15), vowel accuracy (5/15), and articulation via percentile score (9/15). Hesketh et al. (2004), Bird et al. (1995), Nathan et al. (2004), Rvachew et al. (2006–2007), and Mortimer et al. (2008–2010) evaluated PCC from single‐word production on phonology tests rather than connected speech samples. Two case studies investigated prosody: Le Normand et al. (2000) reported appropriate prosody in their participant at both assessment points, while Turner et al. (2019) used a Pairwise Variability Index demonstrating continued difficulties with equal and excess stress into adolescence.

### 3.8 Other Outcomes

Four of 15 studies had literacy outcomes as a primary aim, and 5/9 cohort studies analysed speech, language, and/or phonological awareness skills as a predictor for future literacy difficulties. One study's primary aim was diagnostic (Shriberg et al. 2019); however, the study reported a fourth aim to evaluate persistence and resolution of speech in children retrospectively diagnosed with speech motor delay.

## 4 Discussion

This review examined long‐term speech and related outcomes in 15 studies of children with moderate‐to‐severe SSDs. Given the well acknowledged lack of consensus over speech severity in the speech pathology field, unsurprisingly the definition of ‘moderate’ to ‘severe’ varied across studies (e.g., Lewis et al. 2006 determined speech to be severe if participants scored under the 10th percentile on GFTA and a PCC <90%, while Bird et al. 1995 defined severe as a PCC <50%). While some consistency was observed, especially in case studies via measures of intelligibility or core motor speech features such as syllable integrity, vowel accuracy, and word consistency; even the methods of assessing these features, and determining their severity/impact, were variable.

A lack of consistency and clarity in reporting diagnostic methods and severity inhibits our ability to track the natural history or longitudinal outcomes of SSD. Without clear diagnostic parameters and methods for long‐term evaluation, research lacks an anchor needed to accurately communicate prognosis, treatment efficacy, and guidance for families.

### 4.1 Case Series versus Cohort Studies

Key differences existed between the structure, aims, and findings of case and cohort studies. Cohort studies primarily focussed on predicting long‐term impact and deficits, mostly concerning literacy, while case studies typically documented speech‐specific outcomes over time.

Case studies generally provided clearer speech diagnoses at baseline. While more lenient regarding additional neurodevelopmental diagnoses, the nature and severity of the SSD was largely homogenous (in this review, most case studies documented a primary diagnosis of CAS). Comparatively, cohort studies had strict exclusion criteria for additional neurodevelopmental or oral motor difficulties but included wider ranges of SSD severity that were often difficult to differentiate and interpret. For example, Bird et al. (1995) described participants as having ‘phonological impairment’, but with substantial baseline variability (48% cohort PCC <50%, 29% PCC 51%–64%, 23% PCC 65%–85%), see Table 2. These participants were initially categorised by absence/presence of additional language difficulties, rather than speech severity. Similarly, the five studies from Rvachew et al. (2006–2007) and Mortimer et al. (2008–2010) specified ‘a primary diagnosis of speech delay’ (Rvachew et al. 2007, p. 61) for inclusion, noting children with CAS were not excluded. However, there was no separate follow ‐up or subset analysis of these participants.

Cohort studies often separated participants into speech‐only versus speech‐and‐language disorder groups. However, most speech‐and‐language groups demonstrated significantly lower speech scores, suggesting persistent challenges in speech production which were not further elucidated. It was often in the discussion alone that cohort studies acknowledged a disparity in results between severe, moderate, and mild speech features, especially with regards to increased risks for literacy development. Future longitudinal studies should clearly categorise children by speech severity and diagnosis or at very least underlying mechanism of the impairment (e.g., phonological versus motor) to provide further insight into the natural history of moderate‐to‐severe SSD.

Unsurprisingly, cohort studies typically included wider age ranges at assessment points (e.g., Lewis et al. 2006, T1: 3–7y, T2:7–12y), while case studies had tighter age ranges (participants in n>1 case studies were within 1 year of each other). Again, this speaks to the direct clinical relevance of the case studies to describe natural history with greater accuracy, with the trade‐off of reduced power to comment on speech trajectory at a broader level. Case studies included in this review helped identify longitudinal methods that could be applied at scale in cohort studies, including providing specificity of speech diagnosis from the outset, taking regular repeated measures, and more granular speech specific measurements to thoroughly document changes in speech outcome that are missing in larger cohort studies at this time.

### 4.2 Measuring Severity and Diagnostic Criteria

The scarcity of motor speech follow‐up studies was of note. Most studies (that did not pass full text screening) reported to not actively ‘exclude’ motor SSDs, implying their ‘severe’ participant groups encompassed individuals with apraxia, dysarthria or at least motor speech features. Yet they were not specifically described. Hence this rare population is poorly documented longitudinally, limiting accurate and meaningful counselling to families with the children reportedly most vulnerable to persisting speech and speech‐related difficulties (Mabie and Shriberg 2017; Wren et al. 2023). Shriberg et al. (2019) added nuance by differentiating idiopathic SSD, and severe motor‐based SSDs, introducing ‘speech motor delay’ (SMD), characterised by a delay in neuromotor precision stability (measured by the precision stability index; Wren et al. 2021). While SMD was classified as the least severe of the motor SSDs under this classification, SMD showed persistence beyond 9‐years old in 3/14 participants, suggesting a meaningful clinical subtype. Lewis et al. (2006) also addressed the utility of further subtyping for SSD. Factor analysis found independence between articulation/phonology versus semantic/syntactic disorder patterns in early childhood SSD where articulation/phonological challenges increased risk of spelling and non‐word decoding challenges. Higher scores on articulation/phonological factor scores were associated with greater likelihood of resolved SSD at school age. Newbold et al. (2013) included participants with ‘severe and persisting speech difficulties’ identified as participants with both speech and language difficulties (persisting speech‐only were excluded from follow‐up analysis), positing that this combined profile predicted more severe and persistent errors. The impact of different errors on intelligibility, and speech naturalness/appropriateness is also a consideration. For example, persistence of cluster reduction or vowel errors may pose an increased burden to intelligibility, while persistence of gliding errors may isolate an older child socially due to a more immature sounding speech output. This also targets studies that seek to understand what constitutes as ‘persistent’ SSD. ‘Persistent SSD’ was not included as an interchangeable term for severe SSD in this review, as in many studies it was still unclear whether the speech errors were in keeping with a severe diagnosis (Wren et al. 2023). Studies often did not subtype participants by level of speech severity, but determined participants as persistent (defined as errors persisting beyond the age of typical speech acquisition) or resolved at the follow‐up time point. It is therefore unknown how these children presented initially, apart from having a SSD or whether their persistence is indeed in keeping with a severe phenotype, or the persistence of milder errors, such as gliding. This addresses the need for consistent and collaborative efforts within the research and clinical space to report speech outcomes with consistent terms and standards.

### 4.3 Literacy and Academic Outcomes

Hesketh et al. (2004) found no significance between preschool speech severity (measured by PCC) and school‐age literacy scores. This aligns with Dodd et al. (1995) and Larrivee et al. (1999) position that type of SSD, rather than severity, is more important for predicting academic outcome. Importantly, these results are inconsistent across studies. Larrivee et al. (1999) and Gallagher et al. (2000) found significant relationships between speech production and later reading skills, when measured with non‐word repetition (versus PCC in Hesketh 2004; Eadie et al. 2015; Dodd et al. 2025). This inconsistency again posits the significance of consensus for accurate and reliable measures of speech change. Notably, non‐word repetition is commonly used to assess phonological memory (Eadie et al. 2015; Dodd et al. 2025); however, in younger children this task also taxes articulation abilities and exposes core segmentation difficulties in children with motor‐speech deficits (American Psychiatric Association 2013; McKinnon et al. 2007). Different speech measures identified different predictive features, potentially reflecting distinctions between ‘SSD’ and ‘speech sub‐type’. There was consensus that phonological awareness is most predictive of later literacy, emphasising the importance of ongoing measurement and targeted phonological awareness therapy for children with severe SSD. Lewis et al. (2006) found articulation/phonology factors influenced later reading acquisition through phonological awareness and processing, while sematic/syntactic factors influenced decoding and comprehension—all skills that are vulnerable in children with severe SSDs like CAS.

### 4.4 The General Trajectory

Two cohort studies by the same authors and collected from a larger prospective population study, specifically analysed long‐term speech outcomes in CAS participants, from childhood to adolescence (Lewis et al. 2004, 2023). Lewis et al. (2004) compared the progress of ten CAS participants with speech only, and speech‐and‐language disordered children, from preschool to school age. While most (8/10) CAS participants showed improvement in single‐word accuracy, all experienced persistent difficulties with syllable sequencing, non‐word repetition, and conversation speech errors (particularly voicing, syllable reduction, and vowel errors), as supported by case studies included in this review. While CAS and speech‐and‐language participants showed similar presentations at preschool assessment, follow‐up found CAS participants had pervasive delays in speech, and surprisingly, even poorer language scores than children initially diagnosed with speech‐and‐language disorder. Such broad and pervasive deficits highlight this group to be particularly vulnerable, not only to speech but language and academic challenges, compared to those with mild‐to‐moderate SSD. Later retrospective analysis in Lewis et al. (2023) identified 59% of CAS participants continued to persist at their last assessment (≥12‐years‐old), with 79% participants over 16‐years‐old. Persistence was determined as a percentile score <16% on the GFTA, or ≥4 errors in conversation. In the context of an older age‐group, such scores indicate pervasive difficulties. Preschool scores on the GFTA, syllable repetition, non‐word repetition, and multi‐syllabic word repetition were comparable with resolved CAS participants, while a history of delayed motor milestones, early feeding difficulties and reports of fine or gross‐motor delay significantly differentiated persistent versus resolved groups, suggesting that underlying motor difficulties contribute to persisting challenges. The authors note that despite persistence or resolution status in adolescence, all participants with a history of CAS continued to have difficulties with complex speech production tasks and literacy skills. Persistent difficulties with rhotic vowels, gliding, and stopping, enforces the importance of incorporating complex and later developing sounds into speech therapy.

There was a notable lack of subtypes of moderate‐to‐severe SSDs identified for inclusion in this review, with most being excluded at the full‐text review stage. While the database search did not exclude for other SSDs such as fluency disorders, or dysarthria, papers focussing on fluency or other motor SSDs other than CAS were consequently excluded due to not meeting criteria. Due to the heterogeneity of methods, diagnostic criteria, follow‐up time points, and outcomes across the included peer reviewed studies, no concrete evidence can be communicated to parents and clinicians at this time. As discussed above, another limitation was the lack of clear diagnostic groups, especially in cohort studies. It was often unclear, even in included cohort studies, which participants were initially identified as severe, and if other speech diagnoses were also present.

## 5 Conclusion

Clear recommendations for children and families could not be gleaned from this systematic review examining *the long‐term speech outcomes for children with moderate‐to‐severe SSD*. Findings were constrained due to heterogeneous methodology employed across included studies. Yet our review provides a helpful consolidation of the state of play regarding prognosis for this population at present and many suggestions for future research. Findings highlighted the inherent trade‐off between detailed case studies and larger cohort studies. Whilst clinical case studies provided valuable insights into prognosis and development at the individual level, they could not of course be generalised to inform our understanding of severe SSD outcomes at the population level. Further, larger clinical cohort or population‐based studies did not provide the granularity of data required to reveal specific patterns of change in speech errors over time. This review highlights the need for greater standardisation across assessment procedures, diagnostic terminology and assessment time points for prospective studies examining outcomes for moderate‐to‐severe SSDs. This study highlighted a core clinical research need for more data in this area, as parents consistently ask whether and when their child's moderate‐to‐severe SSD will resolve and we still have too little reliable data available to answer this question. Such data is also urgently needed by health and education organisations struggling to identify data driven effective service delivery models for speech therapy services.

## Funding

This work was supported by the National Health and Medical Research Council (1116976, 1160893).

## Conflicts of Interest

The authors declare no conflicts of interest.

## Supporting information




**Supporting Table 1**: PRISMA 2020 Checklist. **Supporting Table 2**: Appraisal of studies examining long‐term speech outcomes. **Supporting Information Table 3**: Interventions described in 15 eligible studies. **Supporting Table 4**: Summary of five cohort studies, by Rvachew et al., (2006–2010). **Supporting Table 5**: Summary of three case studies by Davis, Marquardt & Jacks (2004–2006). **Supporting Figure 1**: Database search terms (Embase).

## Data Availability

Data available on request from the authors.
