# Supplementary material for: Long‐Term Speech Outcomes in Moderate‐to‐Severe Childhood Speech Sound Disorder: A Systematic Review
Source: Int J Lang Commun Disord. 2026 Apr 5;61(3):e70231. doi: 10.1111/1460-6984.70231 (PMC13051052; doi:10.1111/1460-6984.70231)

**Supplementary Information**

Supplementary Table 1: PRISMA 2020 Checklist

| **Section and Topic** | **Item #** | **Checklist item** | **Location where item is reported** |
| --- | --- | --- | --- |
| **TITLE** | | |  |
| Title | 1 | Identify the report as a systematic review. | p1 Title |
| **ABSTRACT** | | |  |
| Abstract | 2 | See the PRISMA 2020 for Abstracts checklist. | P2 |
| **INTRODUCTION** | | |  |
| Rationale | 3 | Describe the rationale for the review in the context of existing knowledge. | p4-5 |
| Objectives | 4 | Provide an explicit statement of the objective(s) or question(s) the review addresses. | p5 |
| **METHODS** | | |  |
| Eligibility criteria | 5 | Specify the inclusion and exclusion criteria for the review and how studies were grouped for the syntheses. | p6-7 |
| Information sources | 6 | Specify all databases, registers, websites, organisations, reference lists and other sources searched or consulted to identify studies. Specify the date when each source was last searched or consulted. | P6 |
| Search strategy | 7 | Present the full search strategies for all databases, registers and websites, including any filters and limits used. | P6 |
| Selection process | 8 | Specify the methods used to decide whether a study met the inclusion criteria of the review, including how many reviewers screened each record and each report retrieved, whether they worked independently, and if applicable, details of automation tools used in the process. | p7-8 study selection & data extraction |
| Data collection process | 9 | Specify the methods used to collect data from reports, including how many reviewers collected data from each report, whether they worked independently, any processes for obtaining or confirming data from study investigators, and if applicable, details of automation tools used in the process. | P6-7 |
| Data items | 10a | List and define all outcomes for which data were sought. Specify whether all results that were compatible with each outcome domain in each study were sought (e.g. for all measures, time points, analyses), and if not, the methods used to decide which results to collect. | P9 |
|  | 10b | List and define all other variables for which data were sought (e.g. participant and intervention characteristics, funding sources). Describe any assumptions made about any missing or unclear information. | P7 |
| Study risk of bias assessment | 11 | Specify the methods used to assess risk of bias in the included studies, including details of the tool(s) used, how many reviewers assessed each study and whether they worked independently, and if applicable, details of automation tools used in the process. | P7 quality appraisal |
| Effect measures | 12 | Specify for each outcome the effect measure(s) (e.g. risk ratio, mean difference) used in the synthesis or presentation of results. | NA |
| Synthesis methods | 13a | Describe the processes used to decide which studies were eligible for each synthesis (e.g. tabulating the study intervention characteristics and comparing against the planned groups for each synthesis (item #5)). | P7-9 |
|  | 13b | Describe any methods required to prepare the data for presentation or synthesis, such as handling of missing summary statistics, or data conversions. | NA |
|  | 13c | Describe any methods used to tabulate or visually display results of individual studies and syntheses. | P9 CASP |
|  | 13d | Describe any methods used to synthesize results and provide a rationale for the choice(s). If meta-analysis was performed, describe the model(s), method(s) to identify the presence and extent of statistical heterogeneity, and software package(s) used. | P9 CASP |
|  | 13e | Describe any methods used to explore possible causes of heterogeneity among study results (e.g. subgroup analysis, meta-regression). | NA descriptive only |
|  | 13f | Describe any sensitivity analyses conducted to assess robustness of the synthesized results. | NA |
| Reporting bias assessment | 14 | Describe any methods used to assess risk of bias due to missing results in a synthesis (arising from reporting biases). | P9 |
| Certainty assessment | 15 | Describe any methods used to assess certainty (or confidence) in the body of evidence for an outcome. | NA |
| **RESULTS** | | |  |
| Study selection | 16a | Describe the results of the search and selection process, from the number of records identified in the search to the number of studies included in the review, ideally using a flow diagram. | P8-9 + figure 2 for PRISMA diagram |
|  | 16b | Cite studies that might appear to meet the inclusion criteria, but which were excluded, and explain why they were excluded. | P8 |
| Study characteristics | 17 | Cite each included study and present its characteristics. | Table 2, 3 |
| Risk of bias in studies | 18 | Present assessments of risk of bias for each included study. | P8 + supp table 4 |
| Results of individual studies | 19 | For all outcomes, present, for each study: (a) summary statistics for each group (where appropriate) and (b) an effect estimate and its precision (e.g. confidence/credible interval), ideally using structured tables or plots. | Table 2,3 |
| Results of syntheses | 20a | For each synthesis, briefly summarise the characteristics and risk of bias among contributing studies. | P8-9 + supp table 4 |
|  | 20b | Present results of all statistical syntheses conducted. If meta-analysis was done, present for each the summary estimate and its precision (e.g. confidence/credible interval) and measures of statistical heterogeneity. If comparing groups, describe the direction of the effect. | NA descriptive only |
|  | 20c | Present results of all investigations of possible causes of heterogeneity among study results. | P11-15 |
|  | 20d | Present results of all sensitivity analyses conducted to assess the robustness of the synthesized results. | NA |
| Reporting biases | 21 | Present assessments of risk of bias due to missing results (arising from reporting biases) for each synthesis assessed. | NA |
| Certainty of evidence | 22 | Present assessments of certainty (or confidence) in the body of evidence for each outcome assessed. | NA |
| **DISCUSSION** | | |  |
| Discussion | 23a | Provide a general interpretation of the results in the context of other evidence. | P15 |
|  | 23b | Discuss any limitations of the evidence included in the review. | P16 case series vs cohort studies, P18 measuring severity, P20 |
|  | 23c | Discuss any limitations of the review processes used. | P22 conclusion |
|  | 23d | Discuss implications of the results for practice, policy, and future research. | P22 conclusion |
| **OTHER INFORMATION** | | |  |
| Registration and protocol | 24a | Provide registration information for the review, including register name and registration number, or state that the review was not registered. | P6 methods |
|  | 24b | Indicate where the review protocol can be accessed, or state that a protocol was not prepared. | P6 methods |
|  | 24c | Describe and explain any amendments to information provided at registration or in the protocol. | NA |
| Support | 25 | Describe sources of financial or non-financial support for the review, and the role of the funders or sponsors in the review. | P1 Title page |
| Competing interests | 26 | Declare any competing interests of review authors. | P1 Title page + DOC form |
| Availability of data, code and other materials | 27 | Report which of the following are publicly available and where they can be found: template data collection forms; data extracted from included studies; data used for all analyses; analytic code; any other materials used in the review. | P1 Title page |

*From:*  Page MJ, McKenzie JE, Bossuyt PM, Boutron I, Hoffmann TC, Mulrow CD, et al. The PRISMA 2020 statement: an updated guideline for reporting systematic reviews. BMJ 2021;372:n71. doi: 10.1136/bmj.n71

| Supplementary Table 2: Appraisal of studies examining long-term speech outcomes | | | | | | | | | |  | |  |  | |  | |  |  |
| --- | --- | --- | --- | --- | --- | --- | --- | --- | --- | --- | --- | --- | --- | --- | --- | --- | --- | --- |
| **Study** | **Covidence** | |  | | | |  | |  | **CASP** | | | | | |  |  |  |
|  | **Sample size** | **Sample type** | | **Diagnostic group** | **Methods - timeline** | **Methods - assessment** | | **Outcomes** | | **Type** | **Focussed issue?** | | | **Acceptable cohort recruitment?** | | | **Exposure accurately measured?** | |
| Lewis et al., 2004 | n=10 CAS | clinical sample | | dx groups clarified, no severity metric | Time points >6m apart | ax consistent across time-points, standardised ax | | changes over time in scores between groups | | COHORT |  | | |  | | |  | |
| Hesketh 2004 |  | clinical sample, participants in previous Ix study Hesketh 2000 | | mod-sev SSD reported,no dx criteria. Baseline PCC and PA in previous study only. | time points >6m apart | ax consistent across time-points, PCC from standardised ax | | correlation analysis & multiple regression to predict PA & literacy at f/u | | COHORT |  | | | Y recruited from previous Ix study by same authors | | | outlined in previous study only, mod-sev SSD <85 EAT, PCC mod-sev. Sev for f/u sub-sample not reported | |
| Stackhouse 1992 | case study n=2 | Recruitment method unk. n=10 controls recruited from local playgroup | | ax and dx criteria unk. | distinct time points >6m apart | non-standardised speech tasks, pictures developed | | PWC all conditions. Compared with matched articulation age control | | CASE |  | | | Recruitment method unk. Cases defined with detail, described matched controls selection | | | No standardised baseline ax for dx of DAS. Met cluster of features with no physical/neurological changes | |
| To & McLeod 2022 | N=82, n=42 no Ix | initially recruited from population cohort, n=82 with SSD | | Clear dx criteria (HKCAT score under -1.25 SD), no severity measure | time points ~6m intervals for 2.5 years | Same standardised ax at all time points | | survival analysis with binary outcome of word-initial consonant inventory, error, stimulability, intelligibility, EL | | COHORT |  | | |  | | |  | |
| Jacks, Davis, Macquardt 2004-2006 | N=3 males | clinical sample recruited from past sample of children referred for CAS dx | | From Davis 1998 criteria - cluster of features, nil standardised test | time points >6m apart | Artic. tests and C/V productions measured at 3 time points, ax varied between f/u | |  | | CASE |  | | |  | | | Y dx of CAS group via Davis 1998 criteria. Other conditions detailed. Ix prior and during study reported | |
| Shriberg 2019 | n=14 with SMD with f/u available | clinical sample, met criteria for SMD on ≥1 occasion <6y | | met criteria for SMD (PSI <70%), conversation sample available <6 y, & met SMD or no- SMD on ≥1 later recording date | required initial recording to be available <6y, subsequent ax points not reported | retrospective longitudinal data from collaborative research, audio-recorded conversation samples. Similar standards of sampling occurred at each site. | | persistence = meets criteria SMD (PSI >70%). No impact of severity of early SMD associated with persistence. | | COHORT |  | | | Y participants selected from collaborative research at 2 universities with databases of audio-recorded conversation samples. Participants assented to ≥1 studies for SSD. | | | Y SMD dx with PSI <70%, required ax on ≥1 occasion with conversation sample <6y. | |
| Nathan et al 2004 | n=47 | clinical sample referred from SLPs in London, SL and S only groups | | met criteria for S or SL group based on PCC from standardised ax | time points >6m apart | speech tasks not standardised but measured with PCC, PCC also used to measure word and NWR | | low | | COHORT |  | | |  | | | standardised ax, EAT >1SD below mean, severity measured by PCC on EAT average=45.6% | |
| Mortimer & Rvachew 2006-2010 | n=37 but clusters separated into smaller groups | referred to previous larger project by authors at 4-5y, clinical sample referred to SLP | | 4 clusters of dx groups, all <16th percentile on standardised artic. test. | 1-2 f/u points, 1y apart | Same standardised ax across time points, PCC from connected speech sample/picture description task | | report risk of multiple testing across small sample groups | | COHORT |  | | |  | | | required <16^th^ %ile on GFTA, severity determined by GFTA score (only reported in 1/5 studies, uncertain of distribution in other studies with varying sample size) | |
| Pollock 1991 | N=5 but only n=3 f/u | Clinical sample dx at speech & hearing clinic, recruitment method unk. | | met criteria for DAS, severity described | 2 time points | non-standardised word lists, characterised with PVC, PCC, segmental errors, & error patterns | | 84% agreement b/w transcribers, vowel errors significant part of DAS, single-word responses limits understanding of change over time in real-world context | | CASE |  | | | Recruitment methods unk. | | | Subjective ax for DAS dx, standardised artic. ax, sev ax by meeting level 4 on Iowa Severity Rating | |
| Lewis et al., 2023 | n=32 with CAS | recruited from larger 25-y study CFSRS, enrolled in preschool and school age | | met CAS criteria | retrospective analysis, time points unk. | Persistence = <16th percentile on GFTA OR ≥4 speech errors in conversation | | persistence and trajectory of development in CAS | | COHORT |  | | | recruited from CFSRS previously identified with CAS | | | re-evaluated CAS participants to ensure fit CAS dx w/ updated criteria, persistence if <16th%ile in GFTA, and/or ≥4 errors in conversation | |
| Le Normand 2000 | n=1 | referred at 5y6m to St Vincent de Paul Hospital | | No dx criteria reported, description only of neuromotor/cognitive/language outcomes | 2 time points | Speech ax by repetition of 33 words & lexical diversity (number of word in 20 min play session), word productions normed w/ Chevrie Muller et al., 1981 | | Same ax at f/u | | CASE |  | | |  | | | met CAS criteria but no standardised ax at baseline for speech. | |
| Turner et al., 2019 | n=1 | initially assessed by 3rd author at 4y4m & referred to study due to family hx | | met CAS criteria | 17 time points | consistent speech measurements repeated at 3 time points | | standardised ax, speech features & lexical stress via pairwise variability index | | CASE |  | | | referred to related study in research team, data analysed retrospectively | | | dx against ASHA 2007 for CAS | |
| Newbold et al., 2013 | n=4 | clinical sample recruited from original study, compared w/ TD children | | reported with persistent speech and language difficulties, score <2SD | 3 time points but data reported only on T1 and T3. | standardised speech ax, PCC, PWP & PWC | | determined which measurements best identify speech changes over time | | CASE |  | | | selected & analysed after T3 of previous study, determined to have sev. and persisting speech difficulties | | | dx "sev. and persistent speech difficulties" from assumption that SL group have more sev. speech errors. Initial inclusion criteria in past study was >1SD below mean on EAT | |
| Lewis 2006 | n=38 followed from original cohort (n=185) | clinical sample | | mod-sev SSD dx by standardised ax; >3 error types and <90% cut off for conversation sample, no severity | 2 time points, but timelines may differ for each child e.g., T1 4-7y, T2 7-12y | standardised speech ax, conversation speech sample measured with PCC | | primary aim for factor analysis but still fits view for f/u | | COHORT |  | | | referred from clinical caseloads of community SLPs | | | mod-sev SSD determined via standardised ax, severity metric provided | |
| Bird et al., 1995 | n=31 | clinical sample | | picture task not standardised, dx based on PCC (typical PCC >91%) | 3 time points; 7-12m f/u | PCC via picture tasks, same across time points | | PCC, predictions between language and speech sev. on later literacy outcomes | | COHORT |  | | |  | | | non-standardised speech tasks, but items scored for PCC. Sev. metric provided. | |

sev = severe/severity; mod = moderate; ax = assessment; dx = diagnosis; CAS = childhood apraxia of speech; Ix = intervention; PCC = percentage consonant correct; PA = phonological awareness; m = months; y = years; Y = yes; f/u = follow-up; PWC = percentage words correct; SSD = speech sound disorder; EAT = Edinburgh Articulation Test; unk. = unknown; DAS = developmental apraxia of speech; HKAT = Hong Kong Cantonese Articulation Test; SD = standard deviation; EL = expressive language; artic. = articulation; C/V = consonant/vowel; SMD = speech motor delay; PSI = precision stability index; SL = speech and language; S = speech only; NWR = non-word repetition; GFTA = Goldman Fristoe Test of Articulation; %ile = percentile; PVC = percentage vowels correct; CFSRS = Cleveland Family Speech and Reading Study; w/ = with; hx = history; TD = typically developing; SLP = speech language pathologist

| high/N | Unclear/can't tell | low/y |
| --- | --- | --- |
|  |  |  |

| **Study** | **CASP continued** | | |  |  |  |  |  |  | |  |  |  |  |  |
| --- | --- | --- | --- | --- | --- | --- | --- | --- | --- | --- | --- | --- | --- | --- | --- |
|  |  |  |  |  |  |  |  |  |  | |  |  |  |  |  |
|  | **Outcomes accurately measured?** | **Confounding factors identified?** | **Confounding factors - design** | **Was follow-up completed enough?** | **Was follow-up long enough?** | **Results?** | **Precise Results?** | **Do you believe the results?** | **Can results be applied?** | | **Do results fit with past evidence?** | **Implications for practice?** | **Summary - positive** | **Summary - Negative** | **Unknowns** |
| Lewis et al., 2004 |  | unk. impact of Ix | diff. ax at time points mild CAS and CAS w/o lang. impairment excluded | f/u speech | 2y b/w ax | speech changes for each group, groups compared | ANOVAs b/w dx groups for effect size | but only n=10 CAS studied |  |  | | support Ix for lang. and literacy in CAS | standardised ax for baseline & outcomes. Sev. metric provided, | small sample, wide age-range at each ax point | Impact of Ix |
| Hesketh 2004 | PCC measured at f/u but picture task not described. PA measured both time-points (diff tasks) | analysed impact of past Ix study, no impact on current PA |  | f/u speech by changes in PCC | 1 f/u (1y apart) | group level progress in speech, 7/35 persistant T2. No relationship b/w speech sev. preliteracy and literacy at 6-7y. Early PA best predictor of single-word literacy | correlation analysis between Ix and PA. multiple regression analysis for PA, lang. and speech sev. | results sensitive, only 1 child with good PA went on to have low literacy. | Stated “cause or typicality of speech disorder cannot be resolved with the current data, but they underline the need to look at individuals rather than group data " p465 | | most children showed age-appropriate literacy skills at 6-7y. Conflicts w/ Stackhouse (2000), Larivee and Catts (1999), and Gallagher (2000). Noted speech measure different (NWR) | No analysis of persistent speech at T2 & literacy. No impact of change in PCC w/ literacy, but authors acknowledge their group may have more motor phenotype. PA Ix before school recommended. NWR as a PA measure for segmentation and phon processing, disagrees with prior research that uses NWR as speech measure. | speech measures repeated with metric for determining severity | baseline data & methods for speech criteria only in previous study. Unk. picture task or consistent b/w time points. While NWR is often thought to assess phon memory, in younger children this task also taxes artic. | Sev. range & mean of n=35 subgroup at T1, only provided for larger sample from previous study. |
| Stackhouse 1992 |  | n=2 + matched controls | Ix prior to and during study |  |  | speech improved after 4y, same speech errors &. difficult with novel material | Descriptive only | Y | Small sample | | Y |  | Compararison with artic. controls | Small sample | N/A |
| To & McLeod 2022 |  | identified and separated participants with and w/o Ix | acknowledges small sample size, 39 Ix group had significantly more atypical errors and smaller consonant inventory at T1. | same ax for each f/u | every 6m for 2y | time to normalisation shorter for no Ix vs Ix group. Stimulability & intelligibility best predictor of normalisation. | CI provided for medium time to normalisation across variable groups. 3-layer reliability check for transcription | Sev. not defined. Ax measures often used dichotomous definition e.g. ICS-TC cut-off may have participants be typical but v. borderline |  | | contradicts w/ research emphasising predictive factors of atypical speech errors |  | standardised ax for baseline & outcomes. Regular f/u, specified Dx criteria and separation of Ix vs no Ix groups | Sev. at baseline & f/u not reported, dichotomous definitions may not capture those at borderline risk, nature of change (e.g., how atypicality improved/changes, how stimulability changed not reported) | Sev. of group, range of scores, description of change over time |
| Jacks, Davis, Macquardt 2004-2006 |  |  |  |  |  | ++ variability b/w participants. Vowel consistently improved but pattern not uniform between and within participant trajectories | sample too small | Y | Small sample | | Y |  | In depth characterisation over time | Small sample | N/A |
| Shriberg 2019 | Conv. sample measured PCC, II, PVC, PSI criteria. Persistence = meet criteria for SMD after 6y, & consonant deletions/ substitutions that persist beyond 9y. | each child had Ix for SD from preschool. Unclear of impact of Ix on normalisation by 9y. | Different quality of conversation samples between study authors and university procedures. Study participant data collected over several decades. Identify limits of retrospective analysis. | f/u data N/A for 3 participants. Otherwise same data at each recording. Ages/nature of progress not reported | individual f/u points not reported | persistence 3/14, 11/14 resolved <9y. Nil associated btw PSI and persistence. | noted agreeement btw demongraphic of larger cohort (50) and n=14 extracted for retrospective longitudinal analysis. Range and SD provided for all speech measruments. 4 transcriptionists analysed the speech samples for narrow phonetic transcription, prosody-voice and acoustic analysis with mean agreement of 87.9% |  |  | |  |  | standardised mx used throughout, clear dx criteria | f/u time poitns not reported, changes in speech not reported just persistence or resolved, 3/14 nil data bew 6-9y which was required to note presence of persisting difficulty. Uncertain as to whether data from Table 6 is f/u or from initial sample recording for SMD vs no-SMD groups. Assessed for PCC, PVC and intelligibility, longitudinal data on how these measures changed over time were not reported. | individual f/u points, ages at f/u, do not know if persistant SMD cases would have improved if provided motor learning principles, do not have info on 3/14 f/u participants. Could not comment on potential association btw phenotype and persistence. |
| Nathan et al 2004 | 20 pictures of common objects, used in prior study of developmental dylexia but not standardised, PCC measured for WR and NWR | matched S and SL children with TD of similar general ability |  | same output tasks at all time points + extedned WR at T2-T3 |  | comparison b/w S and SL groups, sig difference all time points. SL worse than both at all time points, more sev and persisting. | driving force of persistence Unclear for SL group, language, or underlying severity of speech at baseline? | difficulty with splitting into S and SL where SL had more sev speech impairment |  | |  |  | standardised measures for dx, sev. reported at baseline. Measures repeated across all time points, adequate time btw f/u | severity of speech within S and SL groups, underlying impact of speech severity and subtypes rather than additional lang impairment. | picture naming task not specified or standardised |
| Mortimer & Rvachew 2006-2010 | standardised ax: GFTA, PCC | did not take into account impact of severe or motor speech participants in cohort | mentioned did not exclude for CAS but this group was not extracted or evaluated separately at f/u | standardised measures repeated | 2-3 time points, PreK, K, G1 | Group level improvement GFTA%ile scores G1, h/w 56-59% SSD group below norm at G1. |  |  |  | |  | sub-typing SSD by impact on phon processing skills | criteria for dx, standardised ax repeated across time points | Data obtained across 5 papers, some papers leave out info (e.g., methods and population). Slight variation in sample across studies makes understanding spread of severity difficult | |
| Pollock 1991 |  | Unknown nature of therapy outside study. |  |  |  | 2/3 showed changes in vowel system | very small sample size with variability between participants | only n=3 need replication with larger group. Results match previous studies | Small sample | | vowels typically a significant issue for people with CAS |  | In depth characterisation of change over time | Small sample |  |
| Lewis et al., 2023 | GFTA, 10-15min speech sample at each Ax, MRW, NWR, DDK | small sample, underpowered to detect subtle differences b/w Resolved and Persistent groups. No data on Ix | retrospective Unclear time points | standardised measures repeated, retrospectively analysed | retrospective ≥3 time points for Prek, school & adolescence.. | 59% CAS persistant at last ax | "last ax" not an exact age or time point, 79% were 16-25yo. | Y | Y | | Y | Y | standardised measures for dx retrospectively and validated criteria for dx at baseline. | retrospective analysis | f/u time points not clear |
| Le Normand 2000 | repeated lang ax | Therapy not mentioned | impact of French language on criteria for CAS? |  |  |  | only one participant | Y but only one participant |  | | child had unique presentation with appropriate prosody, plus French, descriptive only |  |  |  |  |
| Turner et al., 2019 | GFTA-2 repeated at 3 time points, connected speech and spontaneous speech measured | did not examine correlation b/w Ix and evolution of speech | single descriptive study - literacy in research and clinical settings used diff. ax tool and had variable findings. Limits of CELF for language skills e.g., pragmatic or higher level language, inter-rater agreement not deterined by 2 SLPs raters |  | Multiple speech assessments over 5+ years |  | one participant | fits anecdotal understanding of disorder, h/w other cohort studies emphasise persistent difficulty in literacy, while others found that some participants with most severe disorder go on to be best spellers/readers | Single case | | Minimal cohort data on speech in adolescence |  | In depth characterisation into adolescence. Wide range of skills and faculties followed-up. | Single case, speech assessments ceased after 10y |  |
| Newbold et al., 2013 | retrospectively identified, standardised ax, NWR, connected speech, measured with PWC, PWP, PCC, phon errors | retrospective, Ix not controlled |  | retrospective |  |  | n=4 | PWC improved significantly in 3/4, PWP 3/4 significant change on naming and RWR, PCC all children showed change over time. Phon processes had not changed noticebly in 2 subjects. that may. Phon analysis not effect for tracking progress. |  | | shows general trajectory similar to other studies showing variability between and within participants. Inconsistent and slow progress |  |  |  |  |
| Lewis et al., 2006 | GFTA, 50-word conv sample for PCC, NWR, MWR, speech error analysis | some measures more than a single component e.g., NWR phon memory and artic. Small sample f/u, receptive phon not assessed | overlapping age groups at T1 and T2 |  | 2 time points, T1 3-7y, T2 7-12y | factor analysis, identified two groups at T2, persistant and resolved, GFTA <65 percentile and 3 or more errors, OCC <96 | Y | Y | Y | | Y - extends, while prior studies indicate lang, not speech predicts later lit (nathan 2004), artic/phon factor predicts deficits in reading in other family members | Y - artic/phon related to persistent SSD…greater risk fo reading difficulties | standardised mx and validated critiera for dx at baseline, severity metric, factor analysis to determine sub-groups with different risk of future difficulties. Consistent f/u | broad age ranges at time points, 7yo participants in T1 and T2, may confound findings from f/u. | overlapping and broad age-range. Were 12yo persistant at T2? Or just the 7yo who may just be beyond expected age of resolution. |
| Bird et al., 1995 | speech tasks not standardised but measured for PCC, same task completed across timepoints | word list developed by authors |  | same tasks repated | 7-12m between re-ax | P and PI groups both at T1 had mod-sev disorder. At T3 group had showed sig improvement now mild PCC. |  |  |  | |  |  | metric for severity to determiend spread of subjects at T1. consistent measurements across time points | did not examine influence of speech severity, just P or PI. Noted in reuslts and discussion impact of speech severity at T1 on later literacy, but was not initial goal of study. | improvements in speech across sev-mild groups |

Unk. = unknown; Ix = intervention; diff = different; ax = assessment; CAS = childhood apraxia of speech; w/o = without; lang = language; f/u = follow-up; b/w = between; Y = yes; ANOVA = analysis of variance ; dx = diagnosis; PA = phonological awareness; phon = phonological; m = months; y = year; PCC = percentage consonant correct; II = intelligibility index (ref); PVC = percentage vowel correct; PSI = precision stability index (ref), SD = speech disorder; WR = word repetition; NWR = non-word repetition; N/A = not applicable; SL = speech and language disorder; S = speech only disorder; sev = severe/severity; GFTA = Goldman Fristoe Test of Articulation; PreK = pre-kindergarten; K = kindergarten/preparatory; G1 = grade 1; h/w = however; info = information; MWR = multi-word repetition; DDK = diadochokinesis task; min = minute(s); PWC = percentage words correct; PWP = percentage word proximity; artic. = articulation

|  |  |  |  |  |  |  |  |  |  |  |  |  |  |
| --- | --- | --- | --- | --- | --- | --- | --- | --- | --- | --- | --- | --- | --- |

| High/N | Unclear/can't tell | Low/y |
| --- | --- | --- |

Supplementary Table 3: Interventions described in 15 eligible studies

| Study | Diagnosis | Primary outcomes | Intervention prior/during study |
| --- | --- | --- | --- |
| Lewis et al., 2004 | S, CAS, SL | Speech | CAS noted slow progress in speech therapy prior to study, all children enrolled in speech therapy (duration and type not reported/unknown to authors) |
| Hesketh, 2004 | Mod-sev SSD | Literacy | Sample collected from previous treatment study (Hesketh, 2000). Children underwent 10 therapy sessions following approaches that emphasise production of metaphonologic awareness. Therapy prior to-or-during time of current study not reported/unknown to authors. |
| To et al., 2022 | SSD | Speech | Selected n=83 from larger prospective study, nil intervention prior to screening. 43/83 received no therapy during study period, 39/83 received therapy (external to study authors) during study period. Types/duration of therapy unknown to authors. |
| Shriberg et al., 2019 | Motor speech delay | Speech | Sample received treatment for idiopathic speech delay (prior to study) commenced at preschool age. Treatment histories not consistent between 6 studies (95% high prevalence treatment prior to enrolment, 0% in low prevalence. |
| Rvachew et al., 2006-2010 | SSD | Literacy, phonological awareness | Therapy undergone during time of study unknown to authors |
| Lewis et al., 2006 | Mod-sev SSD | Speech & literacy | Enrolled in speech therapy at time of study, type/duration unknown to authors |
| Lewis et al., 2023 | CAS | Speech | All participants received speech therapy during study, type/duration unknown to authors. |
| Bird et al., 1995 | Expressive phonological impairment with/without language impairment | Literacy | All children were receiving regular speech therapy when first recruited majority continued therapy throughout study period, type/duration unknown to authors. |
| Jacks, Marquardt, & Davis 2004-2006 | CAS | Speech | P1 commenced therapy at 2y2m, continued throughout study,  P2 commenced therapy at 2y6m with focus on vowel and consonant accuracy  P3 commenced therapy at 4y2m with focus on vowel and consonant accuracy. |
| Pollock & Hall, 1991 | DAS | Speech | Authors report n=2 with more accurate vowel production reported previous treatment for vowel errors. All participants enrolled in intensive summer intervention program at Wendell Johnson Speech and Hearing Clinic (most enrolled for last few years) and received previous therapy including language & articulation. Vowel were a therapy targeted for n=3. Study reports on all therapy for each participant. |
| Newbold, Stackhouse & Wells, 2013 | Severe and persisting speech difficulties | Speech | Summary of speech therapy provided for each participant. 3 of 4 clinic based, variety of intervention types including oral motor, articulation, metaphonological therapy and phonological awareness. |
| Stackhouse & Snowling, 1992 | Developmental Verbal Dyspraxia | Speech | Both participants engaged in 2x weekly speech therapy, focussed on articulation and phonological targets. P1 therapy since 4y (included auditory discrimination and articulation), P2 from 6y (oral sensorimotor, auditory skills, rhythm and phonology). |
| Turner et al., 2019 | CAS | Speech | Regular speech therapy from 3y, fortnightly therapy for ~1 year and attended speech/language programs in prep. Private speech at 6y7m, detailed provided. |
| Le Normand, 2000 | Verbal developmental dyspraxia | Speech | Not reported |
| Nathan et al., 2004 | SSD, SL | Literacy | Referring SLPs reported considerable varaibility in nature and frequnecy of therapy in sample. Children with severe speech received more intensive therapy. Many SLPs were delivering phonological awareness therapy. |

CAS = childhood apraxia of speech; S = speech disorder only; SL = speech and language disorder; SSD = speech sound disorder; mod = moderate; sev = severe; DAS = developmental apraxia of speech.

Supplementary Table 4. Summary of five cohort studies, by Rvachew et al., (2006-2010)

| Study | Shared study characteristics | | Varying study characteristics | | |  |
| --- | --- | --- | --- | --- | --- | --- |
|  | Sample source | Diagnosis | Follow-up period | Participants | Severity | Outcomes |
| Mortimer & Rvachew, 2010 | Participants referred at 4-5 years old from hospital if receiving or waiting to receive speech therapy services | <16^th^ percentile on standardised assessment  Primary diagnosis speech delay  Oral motor exam and hearing within normal limits  English  Did not exclude for concomitant language disorder or childhood apraxia of speech | PreK and end of Grade 1 (2 years between assessment) | N=37  Retrospectively identified 4 clusters for analysis:  1: typical speech and MLU  2: SSD but high MLU both time points  3: SSD low MLU preK, typical grade 1  4: SSD low MLU both time points  Cluster 2-4 with scores below normal limits (?) on standardised measure of artic. accuracy | **PreK clusters 2-4**  GFTA %ile 0.50-14.0  PCC 44.29-91.64  **Grade 1 clusters 2-4**  GFTA %ile 0.50-62  PCC 53.13-98.23 | Expressive language development measured via speech accuracy (GFTA-2), phonological processing, morphosyntactic analysis (PCC and MLU) and expressive language (PPVT) |
| Mortimer & Rvachew, 2008 |  |  | End of PreK and end of K (1 year between assessment) | N=38  PreK 54-66m  K 63-78m | ++ range in GFTA-2 percentile scores  PreK 1-59  K 1-65 | Morphosyntax as a predictor for PA |
| Rvachew, 2007 |  |  | End of PreK and end of Grade 1 (2 years between assessment) | N=17 (SSD low PP),  N=16 (SSD high PP), N=35 (typical)  PreK 53-67m  G1 73-93m | NA | Speech severity and phonological processing as predictive for PA (?) |
| Rvachew, Chiang & Evans, 2007 |  |  | PreK and end of K (1 year between assessment) | N=58 | Used severity ranking from articulation GFTA-2 percentile score  N=20 severe  N=21 moderate  N=17 mild | GFTA-2 for subgrouping of speech errors  PAT depending on speech severity |
| Rvachew, 2006 |  |  | PreK and end of K (1 year between assessment) | N=47  PreK 48-67m  K 61-79m | NA | Speech skills as predictions for literacy (?) |

MLU = mean length of utterance; SSD = speech sound disorder; m=months; PP = phonological processing; GFTA-2 = Goldman Fristoe Test of Articulation – 2^nd^ Edition; PCC = percentage consonant correct; PPVT = ; PA = phonological awareness; PAT; phonological awareness test

Supplementary Table 5. Summary of three case studies by Davis, Marquardt & Jacks (2004-2006)

| Study | Shared study characteristics | |  |  |  | Varying study characteristics |
| --- | --- | --- | --- | --- | --- | --- |
|  | Sample source | Diagnosis | Follow-up | Participants | Assessment | Outcomes |
| Jacks et al., 2006 | N=3 (all males)  4y6m-7y7m | CAS (or DAS prior to 2006) | Yearly follow-up for 3 years | All participants diagnosed with CAS via “cluster” of speech characteristics via Davis 1998.  P1: diagnosed with mild dysarthria and oral apraxia  P2: history of severe RLI (WNL at time of study)  P3: short term auditory memory and metalinguistics difficulties | APP-R, GFTA, KLPA, spontaneous speech sample  Consonant accuracy  Vowel accuracy | Changes in consonant and syllable-level error patterns |
| Davis, Jacks, & Marquardt, 2005 |  |  |  |  |  | Vowel error patterns (inventory, accuracy, error type, accuracy by phrase length and syllable complexity) |
| Marquardt et al., 2004 |  |  |  |  |  | Token analysis: token accuracy, stability, and token-token variability |

Y=years; m=months; CAS = childhood apraxia of speech; DAS = developmental apraxia of speech; RLI = receptive language impairment; WNL = within normal limits; APP-R = assessment of phonological processes - revised ; GFTA = Goldman Fristoe Test of Articulation; KLPA = Khan-Lewis Phonological Assessment (Khan & Lewis, 1986)

Supplementary Figure 1: Database search terms (Embase)


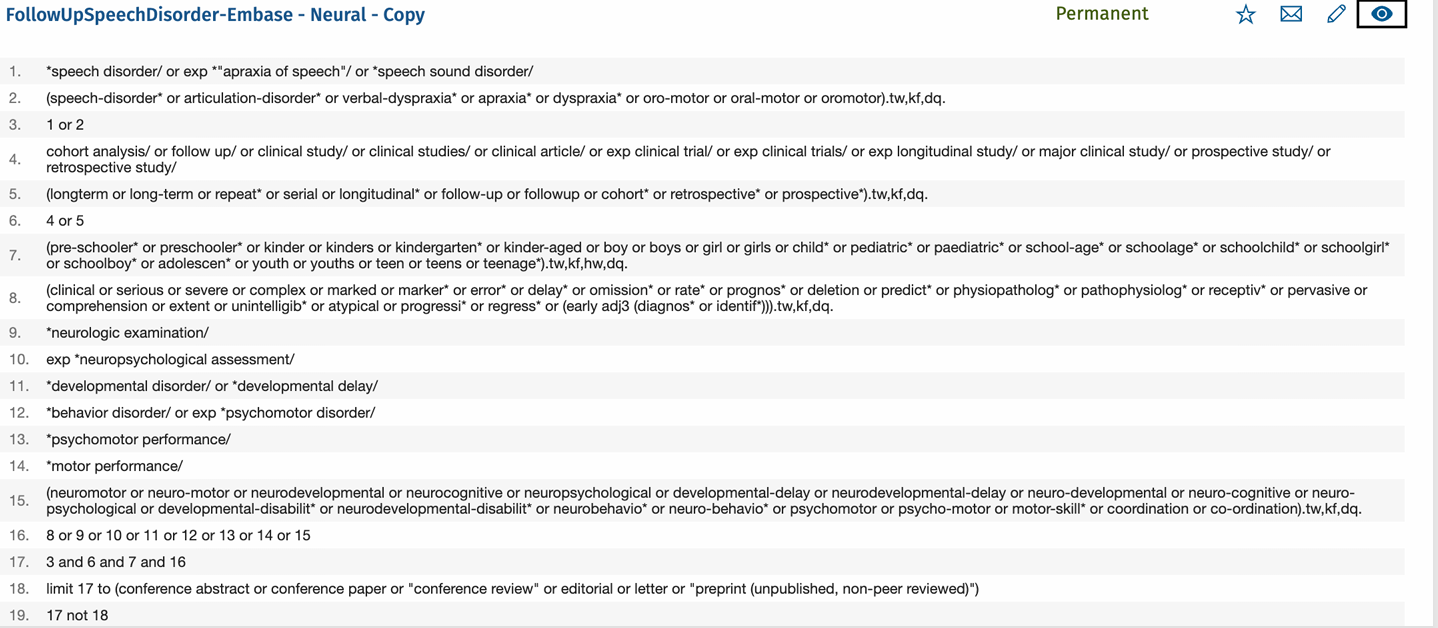

Supplement: Supplementary file 1 — Supporting Table 1: PRISMA 2020 Checklist. Supporting Table 2: Appraisal of studies examining long‐term speech outcomes. Supporting Information Table 3: Interventions described in 15 eligible studies. Supporting Table 4: Summary of five cohort studies, by Rvachew et al., (2006–2010). Supporting Table 5: Summary of three case studies by Davis, Marquardt & Jacks (2004–2006). Supporting Figure 1: Database search terms (Embase). [file JLCD-61-0-s001.docx]
